# Caspase-11-dependent IL-1α release boosts Th17 immunity against *Paracoccidioides brasiliensis*

**DOI:** 10.1371/journal.ppat.1007990

**Published:** 2019-08-19

**Authors:** Natália Ketelut-Carneiro, Camila Oliveira Silva Souza, Luciana Benevides, Luiz Gustavo Gardinassi, Maria Cláudia Silva, Lucas Alves Tavares, Dario Simões Zamboni, João Santana Silva

**Affiliations:** 1 Department of Biochemistry and Immunology, Ribeirão Preto Medical School, University of São Paulo, Ribeirão Preto, SP, Brazil; 2 Department of Clinical Analyses, Toxicology and Food Science, School of Pharmaceutical Sciences of Ribeirão Preto, University of São Paulo, Ribeirão Preto, SP, Brazil; 3 Department of Cell Biology, Ribeirão Preto Medical School, University of São Paulo, Ribeirão Preto, SP, Brazil; 4 Fiocruz-Bi-Institutional Translational Medicine Project, Ribeirão Preto Medical School, University of São Paulo, Ribeirão Preto, SP, Brazil; Memorial Sloan-Kettering Cancer Center, UNITED STATES

## Abstract

The granulomatous lesion resulting from infection with the fungus *Paracoccidioides brasiliensis* is characterized by a compact aggregate of mature cells, surrounded by a fibroblast- and collagen-rich content. Granuloma formation requires signaling elicited by inflammatory molecules such as members of the interleukin-1 family. Two members of this family have been thoroughly studied, namely IL-1α and IL-1β. In this study, we addressed the mechanisms underlying IL-1α secretion and its functional role on the host resistance to fungal infection. We found that, the expression of caspase-11 triggered by *P*. *brasiliensis* infection of macrophages depends on IFN-β production, because its inhibition reduced procaspase-11 levels. Curiously, caspase-11 deficiency did not impair IL-1β production, however caspase-11 was required for a rapid pore-mediated cell lysis. The plasma membrane rupture facilitated the release of IL-1α, which was necessary to induce NO production and restrict fungal replication. Furthermore, *P*. *brasiliensis*-infected macrophages required IL-1α to produce optimal levels of IL-6, a major component of Th17 lymphocyte differentiation. Indeed, IL-1α deficiency accounted for a significant reduction of Th17 lymphocytes in lungs of infected mice, correlating with diminished neutrophil infiltration in the lungs. Strikingly, we identified that IL-1α directly reprograms the transcriptional profile of Th17-committed lymphocytes, increasing cellular proliferation, as for boosting IL-17 production by these cells. Beyond neutrophil chemotaxis *in vivo*, IL-17 also amplified IL-1α production by infected macrophages *in vitro*, endorsing a critical amplification loop of the inflammatory response. Therefore, our data suggest that the IFN-β/caspase-11/IL-1α pathway shapes a protective antifungal Th17 immunity, revealing a molecular mechanism underlying the cross-talk between innate and adaptive immunity.

## Author summary

After infectious insults, the release of intracellular molecules from dying cells, such as IL-1α, provokes a local inflammation as an alert of tissue damage. In this study, we investigated the steps necessary for IL-1α-mediated inflammation and its implications after fungal infection. We identified a molecular mechanism in which IL-1α plays a pivotal role on the control of *Paracoccidioides brasiliensis* infection. We observed that after fungal recognition, macrophages produce IFN-β, a cytokine that promotes the expression of procaspase-11. This enzyme is then activated to trigger a rapid pore-mediated cell lysis, leading to the passive leakage of the cytosolic IL-1α. Once extracellularly, IL-1α functions via paracrine signaling on surrounding cells to enhance the inflammatory response against the pathogen. IL-1α coordinates nitric oxide and IL-6 production by macrophages upon infection, but it also acts directly on CD4^+^IL-17^+^ T lymphocytes by reprograming their transcriptional profile and potentiating IL-17 production. While NO has intrinsic fungicidal properties and IL-6 drives Th17 cell differentiation, IL-17 recruits neutrophils into lungs of infected mice. Furthermore, macrophages synthesize more IL-1α in response to an IL-17-rich milieu. Therefore, IL-1α initiates a sustained and self-perpetuating inflammatory loop that is required for host resistance to *P*. *brasiliensis* infection.

## Introduction

During pulmonary *Paracoccidioides brasiliensis* infection, the granulomatous inflammation is a crucial process to control dissemination and prevent systemic chronic paracoccidioidomycosis (PCM). Concerted efforts of both innate and adaptive immune cells are necessary for fungal recognition and elimination by the host. However, the same mechanisms that destroy the pathogen may also damage the host and exacerbate the disease [1]. Deregulated immunity and tissue remodeling arising from a persistent fungal stimulus are major pathological features of this infection [2]. Resistance to this fungus is primarily mediated by Th1 immunity, while susceptibility is associated with an imbalance towards Th2 response. Nonetheless, cells expressing interleukin-17 (IL-17), such as Th17 lymphocytes, have been detected within and around the granulomas in the skin and oral mucosa lesions from PCM patients [3]. Indeed, IL-17 exerts important roles during *P*. *brasiliensis* infection [4–6].

Macrophages produce diverse pro-inflammatory mediators that initiate and maintain granulomas. Among them, IL-1 signaling has a well-determined function in regulating the recruitment and activation of cells in inflamed tissues [7, 8], but the exact contribution of different members of the IL-1 superfamily to this process still needs to be elucidated. The IL-1 family is now comprised by 11 members, which exhibit complimentary or distinct biological functions [9, 10]. The most well-studied cytokines from this family, IL-1α and IL-1β, bind to the type I IL-1 receptor (IL-1RI) [11], but differ in their maturation processes. In contrast to IL-1β, IL-1α does not require proteolytic processing by caspase-1 inflammasome to become biologically active [12], whereby immature IL-1α also binds to IL-1RI [13]. Nevertheless, because pro-IL-1α lacks a signaling peptide that mediates its secretion from the cell, inflammasome activation can promote IL-1α release under certain circumstances, mostly as a result of cell death [14, 15]. The inflammasome-mediated cell death, termed pyroptosis, is characterized by an immediate pore formation in the cell membrane that increases its permeability [16, 17]. Integrity loss of plasma membrane, osmotic potential imbalance, cell swelling, and eventual bursting is followed by the passive leakage of cytosolic pro-inflammatory molecules from dying cells [18]. Although pyroptosis was initially identified as a caspase-1 inflammasome-dependent process, caspase-11 activation also elicits membrane rupture and cell death [19, 20].

Extracellular release of cytoplasmic components fuels local inflammation as an alert of tissue damage. In fact, bystander cells respond to IL-1α by releasing chemokines and inducing leukocyte infiltration to clear pyroptotic macrophages and avoid excessive inflammation [21, 22]. IL-1α has been shown to play significant roles during fungal diseases [23, 24]. However, the molecular mechanisms that control IL-1α secretion and resistance to pathogenic fungi remain poorly understood. Here, we demonstrate that caspase-11 operates downstream of IFN-β to promote pyroptotic cell death and IL-1α release by bone marrow derived macrophages (BMDMs). IL-1α mediates effective production of nitric oxide (NO) and IL-6, enhancing control of fungal replication by BMDMs. Moreover, this cytokine reprograms the transcriptional profile of Th17 committed lymphocytes, increasing cellular proliferation and IL-17 production. Importantly, IL-1α boosts the Th17 response *in vivo*, promoting adequate neutrophil recruitment into the lung, which reduces fungal burden and confers resistance to the infection. Interestingly, IL-17 stimulation of *P*. *brasiliensis*-infected BMDMs significantly increased IL-1α production, indicating an inflammatory loop between IL-1α and the Th17 response. Collectively, the positive effects of IL-1α over the Th17 response and resistance to *P*. *brasiliensis* infection reveal unrecognized molecular mechanisms at the interface of the innate and adaptive immunity to fungi.

## Results

### Caspase-11 activation promotes pore formation and pyroptosis in response to *P*. *brasiliensis* infection

**Fig 1:**
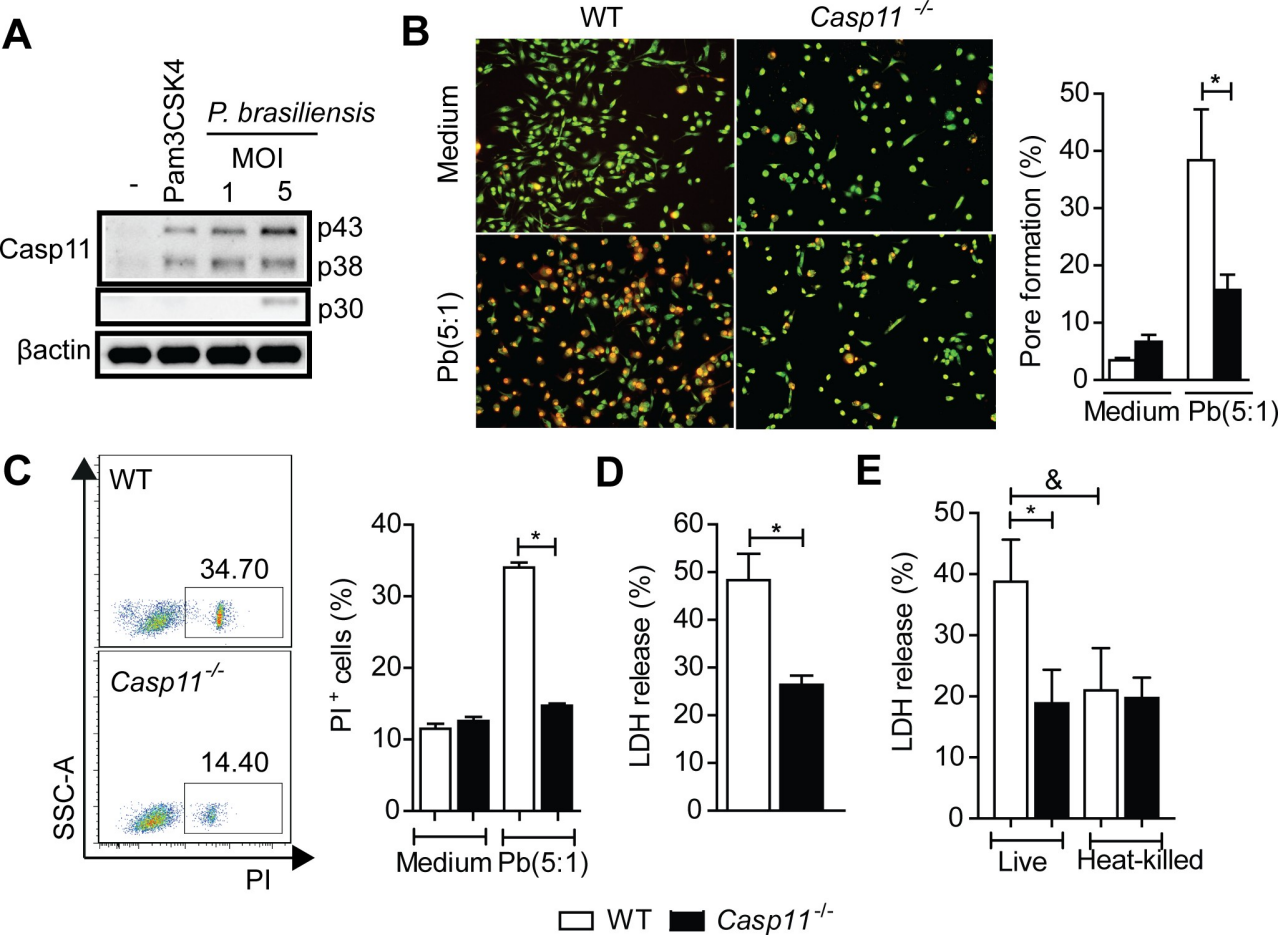
Caspase-11 induces pore formation and pyroptosis in response to *P*. *brasiliensis* infection. **(A)** Caspase-11 expression and activation in cell extract and supernatant, respectively, from WT macrophages infected or not for 24 hours with different MOIs of *P*. *brasiliensis*. Pam3CSK4 stimulation for 3 hours was used as a positive control. **(B)** Fluorescence microscopy of WT and *Casp11*^-/-^ BMDMs stained with acridine orange (green) and ethidium bromide (red) to visualize pore formation after 24 hours of *P*. *brasiliensis* infection. The percentage quantification of cells permeable to ethidium bromide (EtBr) was calculated. **(C)** Propidium iodide (PI) incorporation in either uninfected or *P*. *brasiliensis*-infected WT and *Casp11*^-/-^ BMDMs after 24 hours. **(D)** Lactate dehydrogenase (LDH) was quantified in cell culture supernatants from WT and *Casp11*^-/-^ BMDMs incubated with either live or **(E)** heat-killed *P*. *brasiliensis* (MOI 5) for 24 hours. Each bar represents the mean ± SD of the parameter under analysis. The results are representative of three independent experiments performed in triplicate. Statistical analysis was performed using one-way ANOVA with Tukey’s multiple comparison test (B-C and E-F) or parametric Student’s t test (D). The symbols (*) and (&) indicate statistical difference (p <0.05) compared to the wild type group and live yeasts of *P*. *brasiliensis*, respectively.

Caspase-11-containing inflammasome has been described across a broad range of infections, especially with gram-negative bacteria [25, 26]. To address the molecular mechanisms governing caspase-11 function during fungal diseases, we investigated whether *P*. *brasiliensis* triggers its activation in macrophages. We observed that infected WT BMDMs express both procaspase-11 and cleaved caspase-11, in which the p30 subunit (cleaved caspase-11) was evident in cells infected with a MOI of 5 (**Fig 1A**). Next, we assessed the internalization of the membrane-impermeant dye EtBr to evaluate the requirement of caspase-11 for inducing pores in host cell membranes (pyroptosis). The fungal infection clearly induces EtBr internalization by WT BMDMs, demonstrating that host cells display pores in their membranes after interaction with fungi cells (**Fig 1B**). However, *Casp11*^-/-^ BMDMs were significantly less permissive to EtBr, confirming a requirement for caspase-11 on *P*. *brasiliensis*-induced pore formation (**Fig 1B)**. Using propidium iodide internalization assay and flow cytometry, we also found that the membrane integrity was maintained in BMDMs derived from *Casp11*^-/-^ mice but lost in WT cells (**Fig 1C**). To corroborate that host cell pore-formation and reduced membrane integrity indeed reflect pyroptosis, we quantified LDH levels in cell culture supernatants after fungal infection. Compared to WT BMDMs, LDH levels were significantly reduced in *Casp11*^-/-^ BMDMs culture supernatants (**Fig 1D**). Interestingly, the caspase-11-mediated lysis requires *P*. *brasiliensis* to be alive, once heat-killed yeasts did not stimulate significant LDH release (**Fig 1E**). These results indicate that caspase-11 activation by *P*. *brasiliensis* is essential for pore-mediated cell lysis and pyroptosis.

### IL-1α is released via IFN-β/caspase-11 pathway following fungus infection

**Fig 2:**
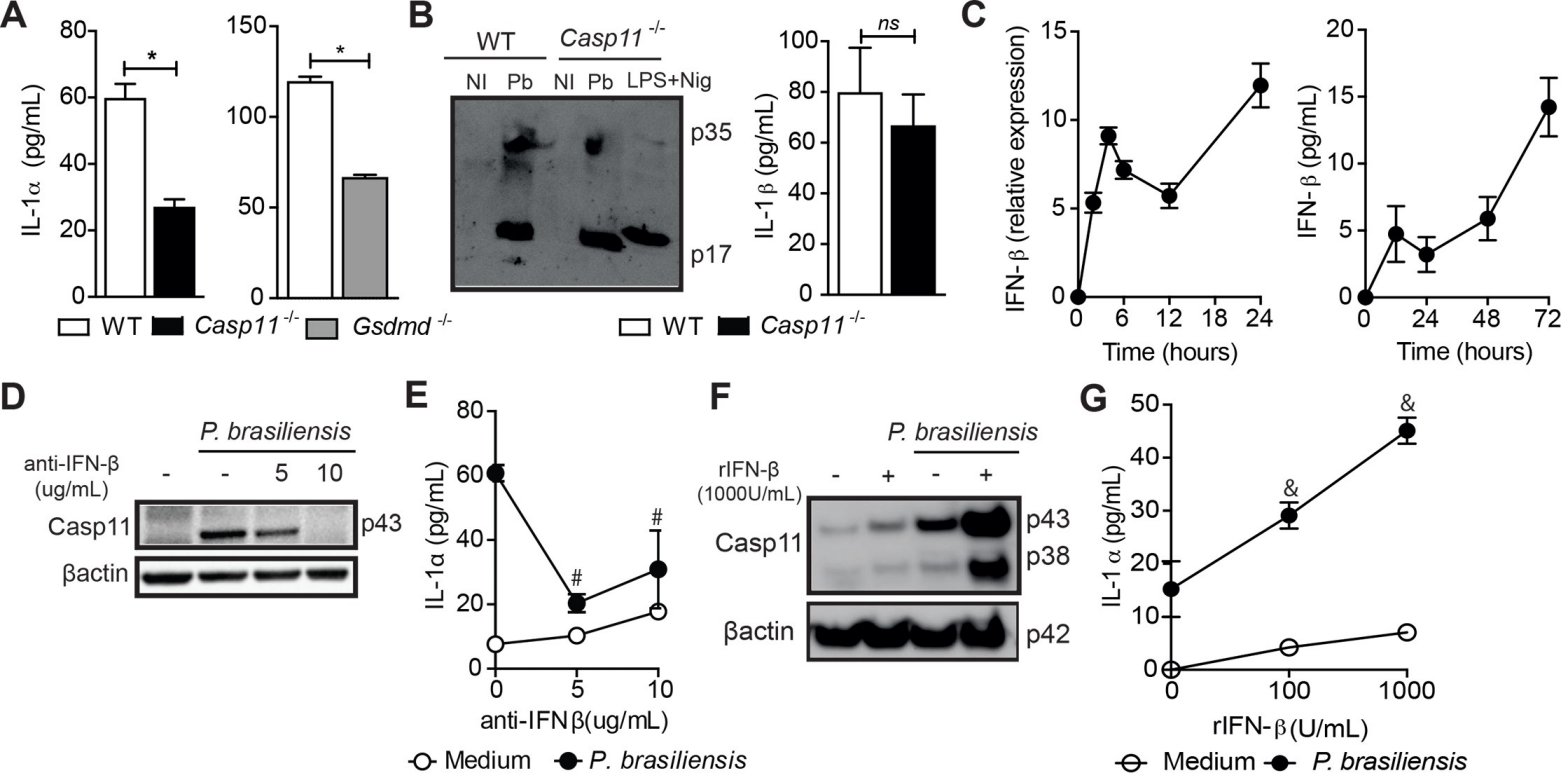
IFN-β-dependent caspase-11 activation mediates IL-1α production by *P*. *brasiliensis*. **(A)** BMDMs from WT, *Casp11*^-/-^ and *Gsdmd*^-/-^ mice were incubated with 5 yeasts per cell and the supernatant was harvested for IL-1α measurement after 48 hours **(B)** IL-1β released by WT and *Casp11*^-/-^ BMDMs after 48 hours of incubation with *P*. *brasiliensis* (MOI 5) was analyzed by western blotting and ELISA. As a control of IL-1β p17 subunit secretion, LPS-primed WT BMDMs were stimulated with nigericin (20uM) for 40 minutes. Combined data from three different experiments were plotted. **(C)** Time-course of IFN-β expression and production in BMDMs after *P*. *brasiliensis* infection. **(D)** Caspase-11 immunoblot and **(E)** IL-1α quantification in WT BMDMs treated with indicated doses of anti-IFN-β at the time of *P*. *brasiliensis* infection. **(F)** WT BMDMs were pre-treated with or without recombinant IFN-β 2 hours before *P*. *brasiliensis* infection. The presence of caspase-11 and **(G)** IL-1α were assayed by immunoblotting and ELISA after 24 and 48 hours, respectively. Data are representative of three independent experiments expressed as the mean of triplicate wells. Statistical analysis was performed using non-parametric Mann-Whitney U test (A), parametric Student’s t test (B) or one-way ANOVA with Tukey’s multiple comparison test (E and G). Error bars depict ± SD. (*) p < 0.05 versus *P*. *brasiliensis*-infected WT mice. (#) p <0.05 compared to infected macrophages that were not subjected to the anti-IFN-β treatment. (&) depicted p < 0.05 comparing *P*. *brasiliensis*-infected samples treated with non-treated. NI: non-infected. ns: not significant.

Next, we investigated whether the fungus-induced caspase-11 activation and host cell pyroptosis are followed by passive IL-1α release. To achieve this, we used BMDMs from mice deficient for caspase-11 and gasdermin D, a 53 kDa protein that generates an N-terminal pore-forming fragment after inflammasome-dependent activation of caspase-1 or -11 [19, 20, 27–29]. We found that IL-1α levels were significantly reduced in cell culture supernatants from both infected *Casp11*^-/-^ and *Gsdmd*^-/-^ BMDMs (**Fig 2A**), suggesting that caspase-11 and pyroptosis are vital to ensure optimal IL-1α release. Caspase-1 and caspase-8 are required for IL-1β processing and secretion during this fungal infection [30, 31]. Thus, we tested whether caspase-11 is also involved in IL-1β release in this context. WT and *Casp11*^-/-^ BMDMs incubated with *P*. *brasiliensis* synthesized and secreted similar amounts of IL-1β, demonstrating that caspase-11 activation is only necessary for IL-1α secretion (**Fig 2B**). Detailed time course analysis of IL-1β secretion by infected WT and *Casp11*^-/-^ cells revealed that caspase-11-deficient BMDMs behave similarly to WT cells. Even pondering different timepoints, IL-1β release occurred in a caspase-11-independent manner (**S1A Fig**), excluding that the IL-1β might peak at a time other than 48 hours. Importantly, levels of IL-1β were similar in the lungs of WT and *Casp11*^-/-^ mice at 15 and 30 dpi, validating the *in vitro* data (**S1B Fig**).

*P*. *brasiliensis* infection of macrophages increased levels of IFN-β over time (**Fig 2C**), whereas IFN-β induces caspase-11 expression and activation [32]. To understand the upstream events of caspase-11 activation, we explored the role of IFN-β during *P*. *brasiliensis*-induced IL-1α release. Incubation of WT BMDMs with IFN-β-neutralizing antibody at the same time of the infection markedly reduced procaspase-11 expression (**Fig 2D**). As expected, IL-1α release was also impaired in these BMDMs (**Fig 2E**). Moreover, caspase-11 expression (**Fig 2F)** and IL-1α release (**Fig 2G)** were strongly enhanced by addition of rIFN-β to the culture 2 hours prior *P*. *brasiliensis* infection. Collectively, the data point to a model where the fungus induces IFN-β production, whose signaling promotes procaspase-11 expression, cleavage and activation of caspase-11, resulting in pyroptosis and IL-1α release to the extracellular environment.

### Caspase-11 controls IL-1α release to limit fungal replication

**Fig 3:**
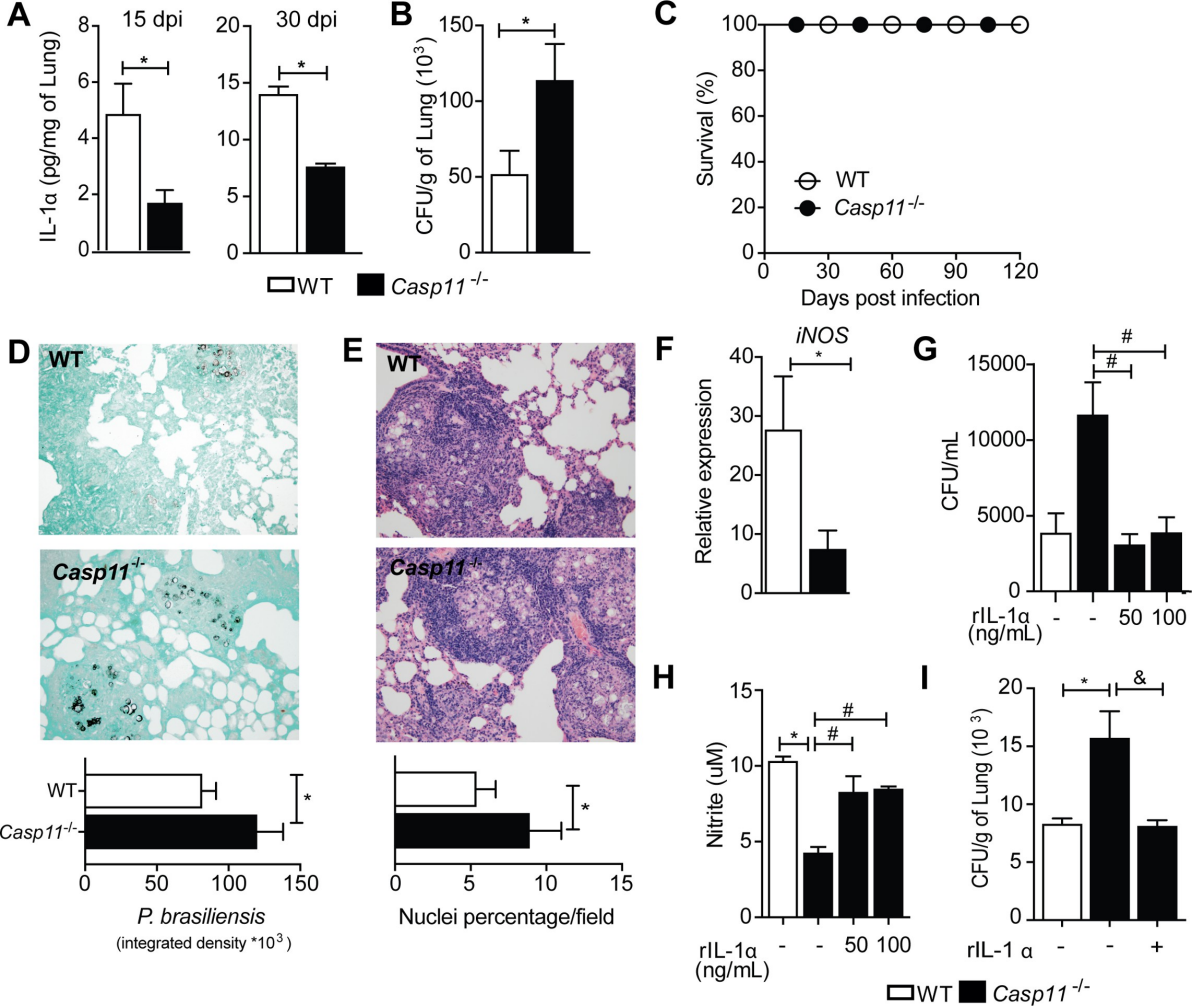
Caspase-11 is required to fungal clearance. **(A)** WT and caspase-11-deficient mice were intravenously infected with 1x10^6^ viable yeasts of Pb18. On the 15th and 30th day of infection, IL-1α was quantified in lungs with ELISA. **(B)** Lung homogenates were diluted and plated in BHI-agar medium for CFU determination according to the tissue weight. **(C)** Wild-type and *Casp11*^-/-^ mice were challenged with 1x10^6^ *P*. *brasiliensis*, and the survival of the Pb18-infected mice was monitored daily for 120 days. **(D)** *P*. *brasiliensis* was identified in the lung of respective mice after Grocott staining (magnification of 200x). Quantitative representation of the *P*. *brasiliensis* integrated density is shown. **(E)** Histological sections of lungs from WT and *Casp11*^-/-^ mice after 30 days after infection with *P*. *brasiliensis*. The granulomatous lesion images were captured under a light microscope after H&E staining and the nuclei percentage per field was evaluated. **(F)** PCR analysis of mRNA expression of iNOS in the lung tissue of WT and *Casp11*^-/-^ mice at 30dpi. β2m was used as a housekeeping gene. **(G)** Fungal growth and **(H)** nitrite levels quantified in WT and *Casp11*^-/-^ BMDMs infected with *P*. *brasiliensis* and cultured or not with increasing concentrations of rIL-1α. **(I)** *Casp11*^-/-^ mice infected i.v. with 1x10^6^ *P*. *brasiliensis* cells were untreated or treated i.n. with rIL-1α (100 ng/animal) at the onset of the infection. After 30 dpi, the CFUs were measured. Error bars show the mean ± SD of 6 mice. Data are representative of three independent experiments. Statistical analysis was performed using log-rank test (Mantel–Cox) (C), non-parametric Mann-Whitney U test (A-B and F), parametric Student’s t test (D-E) or one-way ANOVA with Tukey’s multiple comparison test (G-I). (*) denotes p <0.05 compared to *P*. *brasiliensis*-infected WT mice (C57BL/6). (#) p <0.05 relative to *Il1a*^-/-^ BMDMs not treated with rIL-1α. (&) indicates p <0.05 compared with non-treated *Casp11*^-/-^ mice.

To determine the role of caspase-11 during host responses *in vivo*, we infected WT and *Casp11*^-/-^ mice with *P*. *brasiliensis*. Confirming the findings obtained in cell culture experiments, caspase-11-deficient animals exhibited reduced pulmonary levels of IL-1α after 15 and 30 dpi compared to WT mice (**Fig 3A**). *Casp11*^-/-^ mice also harbored increased fungal burden in the lung at the same time of infection (**Fig 3B**). However, survival of infected *Casp11*^-/-^ mice remained similar to those of WT mice (**Fig 3C**). In accordance, *in situ* Grocott staining of fungi cells showed that infected *Casp11*^-/-^ mice were unable to limit the fungal growth in the lung tissue at 30 dpi (**Fig 3D**). We presumed that elevated fungal burden would be associated with an intense pulmonary inflammation. However, despite the increase in the inflammatory infiltrate, H&E-stained histological sections of infected *Casp11*^-/-^ mice revealed well-organized granulomas with a few diffuse inflammatory reactions in the pulmonary parenchyma (**Fig 3E**). These data demonstrate that, although dispensable for granuloma formation, caspase-11 plays a significant role in the control of *P*. *brasiliensis* infection. Reduced levels of IL-1α in the lungs of infected *Casp11*^-/-^ mice along with the lower expression of inducible nitric oxide synthase (iNOS) (**Fig 3F**), suggest that effector mechanisms downstream caspase-11 activation could be mediated by IL-1α and nitric oxide (NO), an important antifungal compound. Thus, we tested the fungal killing capacity of *Casp11*^-/-^ BMDMs and the effects of exogenous rIL-1α. Compared to WT BMDMs, infected *Casp11*^-/-^ cells exhibited increased fungal loads (**Fig 3G**). However, rIL-1α rescued the fungicide activity of *Casp11*^-/-^ cells (**Fig 3G**), correlating with the increased production of NO (**Fig 3H**). In addition, the administration of recombinant IL-1α to *Casp11*^-/-^ infected mice reversed their pulmonary fungal load, which was similar to that of the WT phenotype (**Fig 3I**). These data indicate that caspase-11 regulates IL-1α release to limit fungal replication by macrophages.

### IL-1α mediates resistance against *P*. *brasiliensis* infection

**Fig 4:**
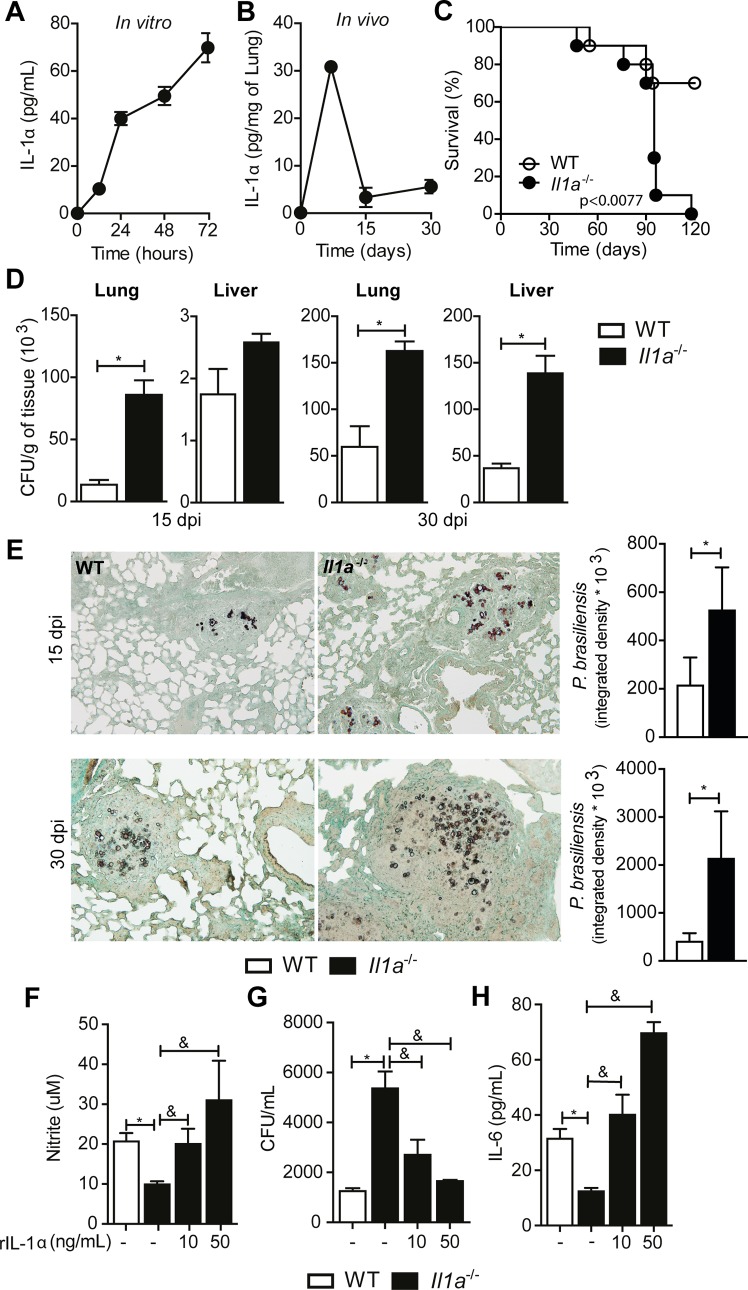
IL-1α protects against infection with *P*. *brasiliensis*. **(A)** Dynamics of IL-1α production *in vivo* and **(B)** *in vitro* after *P*. *brasiliensis* challenge. **(C)** Survival rate of WT and *Il1a*^-/-^ mice intravenously infected with 1x10^6^ yeast forms of Pb18. **(D)** Pulmonary and hepatic fungal load in WT and *Il1a*^-/-^ mice at 15 and 30 dpi with 1x10^6^ *P*. *brasiliensis* cells. **(E)** Lung tissue sections obtained from WT and *Il1a*^-/-^ mice infected with *P*. *brasiliensis* for 30 days were oxidized by chromic acid and subjected to silver impregnation for fungus detection (magnification of 200x) and quantification. Each column represents a mean ± SD of organs collected from six mice. Data are representative of two independent experiments. **(F)** BMDMs from wild-type or IL-1α knock-out mice were pre-activated for 6 hours with recombinant IL-1α (rIL-1α; 10, or 50 ng/mL), followed by infection with *P*. *brasiliensis* in a yeast:macrophage ratio of 5:1. After 48 hours of infection, the levels of nitrite and **(H)** IL-6 in the culture supernatant were measured using the Griess assay and ELISA, respectively. **(G)** Fungal load in BMDMs from WT or *Il1a*^-/-^ mice stimulated with rIL-1α for 6 hours. The results are representative of three independent experiments performed in triplicate. Statistical analysis was performed using log-rank test (Mantel–Cox) (C), non-parametric Mann-Whitney U test (D), parametric Student’s t test (E) or one-way ANOVA with Tukey’s multiple comparison test (F-H). (*) p <0.05 relative to the WT animals (C57BL/6). (&) p< 0.05, compared with IL-1α-treated *Il1a*^-/-^ macrophages. dpi: days post infection.

To better characterize the biological role of IL-1α, we first analyzed the kinetics of IL-1α production over the time of fungal infection. *In vitro*, WT BMDMs initiated IL-1α secretion after 12 h, and increased until 72 h (**Fig 4A**). *In vivo*, pulmonary levels of IL-1α increased in the first 7 days of infection but declined to baseline levels at 15 dpi (**Fig 4B**). Next, we evaluated the biological function of IL-1α in PCM by following survival of *P*. *brasiliensis-*infected WT and *Il1a*^-/-^ mice. Strikingly, 100% of *Il1a*^-/-^ animals succumbed to infection, in contrast to 30% of WT mice (**Fig 4C**). We also recovered more fungal colonies in the lungs, but not livers of *Il1a*^-/-^ animals after 15 days of infection (**Fig 4D**). However, *Il1a*^-/-^ mice showed higher fungal counts in the lungs and livers at 30 dpi (**Fig 4D**). Gomori staining of lung tissue sections confirmed that IL-1α deficiency suppresses pulmonary fungal clearance (**Fig 4E**), providing further evidence for a protective role of IL-1α in the control of *P*. *brasiliensis* infection.

The granulomatous inflammation is a crucial process to control *P*. *brasiliensis* dissemination and prevent systemic disease. IL-1 signaling has been shown to promote efficient granuloma formation [7], which would explain higher susceptibility of *Il1a*^-/-^ mice to the fungal infection. However, caspase-11 does not seem to be important for this process **(Fig 3E)**, suggesting that the caspase-11/IL-1α axis is not critical for the structural formation of granulomas in this context. Indeed, we observed that the elevated number of cells, presented in IL-1α-deficient mice after 15 and 30 dpi, was arranged in organized and well-formed granulomas (**S2A Fig**). Furthermore, *Il1a*^-/-^ animals presented a large number of reticulin fibers delimiting the peripheral area and compartmentalizing the granulomatous structures (**S2B Fig**). These data indicate that the caspase-11/IL-1α pathway promotes resistance to *P*. *brasiliensis* by mechanisms other than enhancing the granulomatous response. Exogenous rIL-1α enhanced NO production and fungal clearance by *Casp11*^-/-^ cells (**Fig 3G and 3H**), showing that IL-1α drives efficient NO production by macrophages in response to *P*. *brasiliensis*. Moreover, BMDMs from *Il1a*^-/-^ mice incubated with *P*. *brasiliensis* produced an average of 19 μM of nitrite, contrasting with the 31 μM produced by WT cells (**S3A Fig**). This data correlated with reduced fungicide capacity of *Il1a*^-/-^ cells (**S3B Fig**). Even after activation with IFN-γ, IL-1α deficiency impaired NO production and yeast clearance (**S3A and S3B Fig**). Importantly, the addition of rIL1-α at 50 ng/mL to *Il1a*^-/-^ cell culture increased NO synthesis by 210% (**Fig 4F**) and reduced fungal burden by approximately 70% (**Fig 4G**). Accordingly, the importance of IL-1α in driving NO production in response to *P*. *brasiliensis* was also evident *in vivo*. Compared to WT animals, there was a down-regulation of *Nos2* gene expression in the lungs of *Il1a*^-/-^ mice (**S3C Fig**). These results show that IL-1α regulates NO production by macrophages, coordinating an important fungicide mechanism.

### IL-1α deficiency mitigates antifungal Th17 immune response

**Fig 5:**
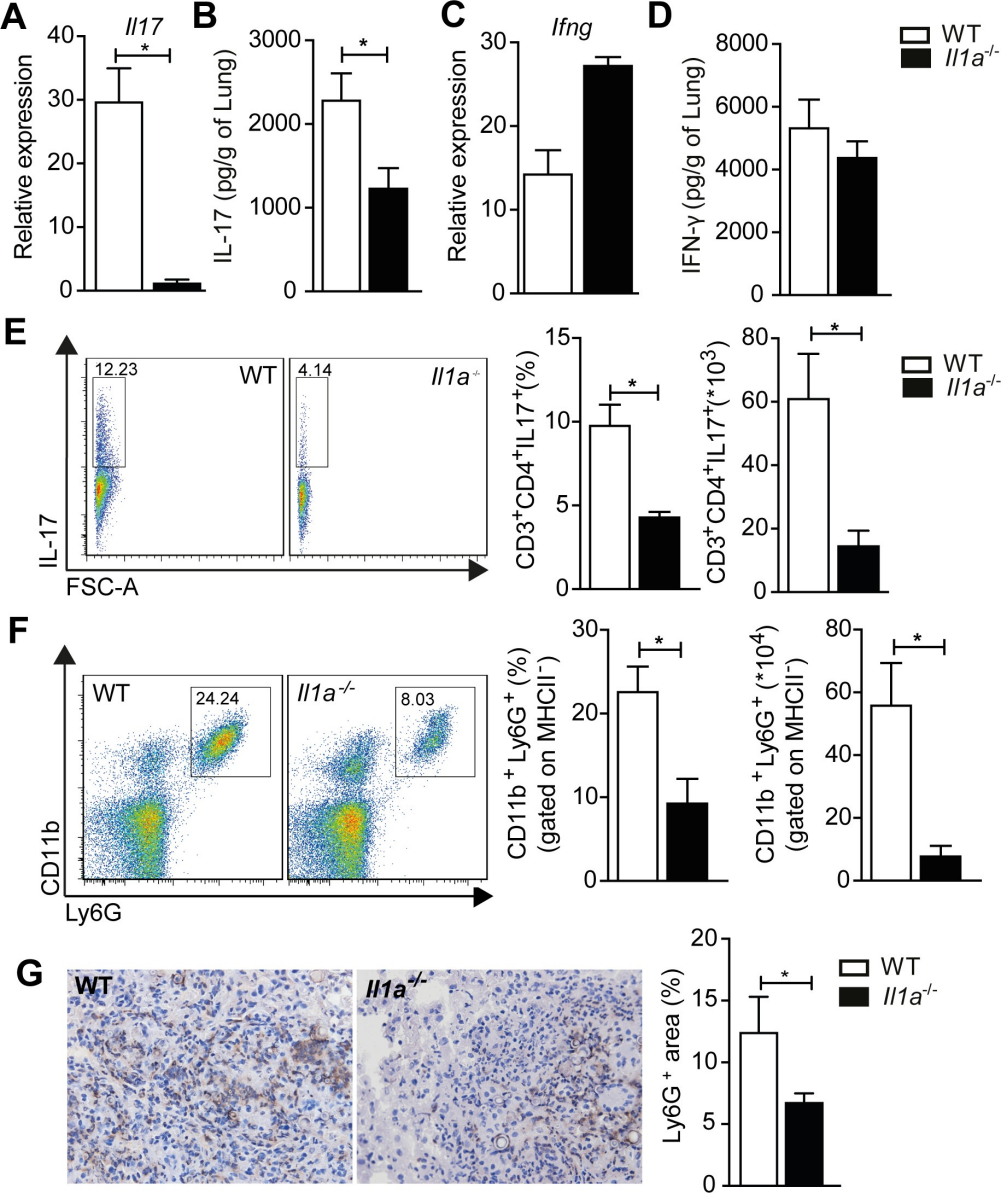
IL-1α boosts antifungal Th17 response during experimental PCM. **(A and B)** Transcriptional and protein levels of IL-17 and **(C and D**) IFN-γ were quantified in lung homogenates from WT and *Il1a*^-/-^ mice challenged for 30 days with viable yeasts of *P*. *brasiliensis*. **(E)** The frequency and absolute number of pulmonary CD3^+^CD4^+^IL-17^+^ T cells were determined by flow cytometry after 30 days of *P*. *brasiliensis* infection of WT and *Il1a* ^-/-^ mice. **(F)** Frequency and the absolute number of MHC^-^CD11b^+^Ly6G^+^ neutrophils were determined by flow cytometry after 30 days of infection of WT and *Il1a*^-/-^ mice with 1x10^6^ yeast cells of *P*. *brasiliensis*. **(G)** Ly6G expression (neutrophil marker) in frozen lung fragments was evaluated by immunohistochemistry reaction at 30 dpi (magnification of 200x). Each column represents the mean ± SD. Data are representative of three independent experiments. Statistical analysis was performed using non-parametric Mann-Whitney U test (A, D-G) or parametric Student’s t test (B-C). (*) p<0.05 knockout mice versus C57BL/6 mice. FSC: forward scatter.

Several studies have described that Th1 and Th17 responses are implicated in the host resistance to *P*. *brasiliensis* [4, 33]. Interestingly, a fungal infection of *Il1a*^-/-^ BMDMs resulted in reduced IL-6 production, whereas exogenous rIL-1α rescued IL-6 levels (**Fig 4H**). Considering the pivotal role of IL-6 for Th17 lymphocyte differentiation [34, 35], we hypothesized that IL-1α deficiency would particularly compromise the Th17 cell-mediated immunity. In fact, infected *Il1a*^-/-^ mice exhibited reduced transcriptional and protein levels of IL-17 (**Fig 5A and 5B,** respectively) but not of IFN-γ (**Fig 5C and 5D**) in lungs. Corroborating this data, *Il1a*^-/-^ animals exhibited reduced frequency and number of IL-17-expressing CD4^+^ T cells in the lung at 30 days post *P*. *brasiliensis* infection (**Fig 5E**). Importantly, infection of *Casp11*^-/-^ mice with *P*. *brasiliensis* recapitulated all aspects of the Th17 response observed in the lung of *Il1a*^-/-^ animals at 30dpi (**S4A–S4C Fig**). Treatment of *Casp11*^*-/-*^ mice with rIL-1α not only reduced CFU counts (Fig 3I), but also increased IL-17 levels in the lung at 30 dpi, supporting a role for this cytokine in boosting the Th17 response (**S4D Fig**). Together, these data strongly support a model in which IL-1α is released due caspase-11 activation and pyroptosis to promote efficient antifungal Th17 immunity.

A key question that arises from these results is whether IL-1α-deficiency impairs Th17 cell migration from mediastinal lymph nodes to the lung. However, we observed that IL17^+^ T cells did not accumulate in the lymph nodes collected from infected *Il1a*^-/-^ mice (**S5A Fig**). In addition, a disruption of the chemoattractant axis CCL20/CCR6 in *Il1a*^-/-^ mice could explain the reduced Th17 response after 30 days of infection. Despite increased transcriptional and protein levels of CCL20 in the lung of *Il1a*^-/-^ animals (**S5B and S5C Fig**), CCR6 expression in Th17 cells was similar between infected *Il1a*^-/-^ and WT mice (**S5D Fig**). Finally, Th17 responses promote a strong neutrophil influx into sites of inflammation [36–38], suggesting that IL-1α deficiency impacts the neutrophilic response against the fungus. Accordingly, we found that decreased IL-17A production correlated with a significant reduction in neutrophil recruitment to the lungs of infected mice (**Fig 5F**). We confirmed this result by showing that *Il1a*^-/-^ animals infected for 30 days express lower amounts of Ly6G (neutrophil surface marker) in the lung tissue compared to WT mice (**Fig 5G**). Collectively, these data demonstrate that caspase-11 and IL-1α are critical for the development of a productive Th17 response to *P*. *brasiliensis* infection.

### IL-1α induces a molecular reprogramming of Th17 lymphocytes

**Fig 6:**
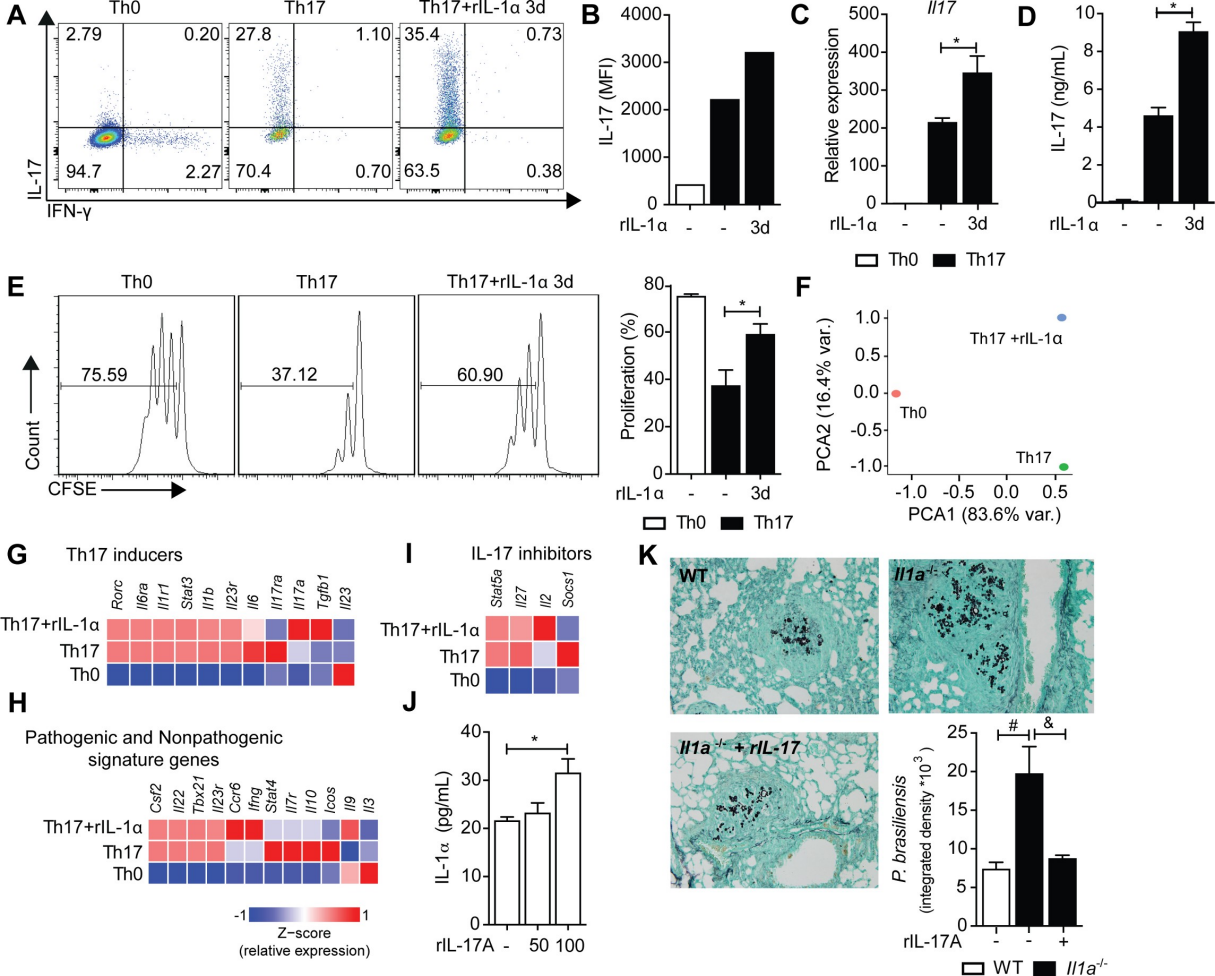
IL-1α induces an enhanced Th17 cell phenotype. **(A)** CD4^+^ T cells from lymph nodes and spleen were isolated by magnetic beads from naïve WT mice and cultured on anti-CD3- and CD28-coated plates in a Th0- or Th17 polarizing conditions for 3 days. Following IL-1α was added or not to the indicated cultures. IL-17- or IFN-γ- expressing cells were measured by flow cytometry after 5 days of PMA and ionomycin stimulation. Values represent the percentage of IL-17^+^ cells **(B)** Mean fluorescence intensity (MFI) of IL-17 in IL-17^+^IFN-γ^-^ cells. Data were obtained by pooling samples from four wells. **(C)** Th17 cells differentiated *in vitro* with TGF-β, IL-6 and IL-23 were incubated or not with IL-1α on the third day. Cell extracts was collected to quantify *Il17* expression. **(D)** IL-17 production was quantified in the cell culture supernatant. **(E)** CFSE labeled CD4^+^ T cells were polyclonally stimulated in the presence of TGF-β, IL-6, IL-23 and either IL-1α or media. The histograms reveal the cell division index after 5 days of culture. **(F)** Principal component analysis of the whole array of Th17-related genes obtained from Th0, conventional Th17 (Th17) and Th17+IL-1α cells by qPCR. The data were obtained by combining samples from four different wells. **(G-I)** Heatmaps illustrating differential gene expression Th17-related genes grouped based on functional activity. (**J**) WT macrophages were incubated for 6 hours with recombinant IL-17A (rIL-17A; 0, 50 or 100 ng/mL) before *P*. *brasiliensis* stimulation, and IL-1α levels were determined by ELISA after 48 hours post-infection. **(K)** Pulmonary fungal load *in situ* at 30dpi in *Il1a*^-/-^ mice non-treated or treated with rIL-17A (500 ng/mice) every three days from day 15dpi. The results are expressed as the mean ± SD using quadruplicate samples from one out of three independent experiments. Statistical analysis was performed using one-way ANOVA with Tukey’s multiple comparison test (C-E and I-J). (*) means p<0.05 compared to Th17 cells that were not treated with IL-1α at the third day of differentiation. (#) means p<0.05 compared to WT mice. (&) means p<0.05 compared to untreated *Il1a*^-/-^ mice.

In view of the significant impact of IL-1α deficiency over the Th17 lymphocyte compartment, we isolated CD4^+^ T cells from WT and *Il1a*^-/-^ mice and cultured them under Th17-polarizing conditions to rule out the possibility that IL-1α directly influences Th17 cell differentiation. In both groups, CD4^+^ T cells differentiated equally well for the Th17 profile (**S6A and S6B Fig**). Hence, we conclude that the decline of IL-17^+^ T cell population in *Il1a*^-/-^ mice was not due an impaired intrinsic capacity to differentiate into Th17 lymphocytes. To understand the immune-modulatory effects of IL-1α over the Th17 response, isolated CD4^+^ T cells were polarized to the Th17 profile, whereby we added rIL-1α to these cells on the 3^rd^ day of differentiation. As a readout for the effectiveness of the Th17 cell differentiation, we confirmed that these cells were unable to produce IFN-γ (**S6C and S6D Fig**). Surprisingly, the addition of rIL-1α elevated the percentage of IL-17 producing cells (**Fig 6A**), also visualized by increased IL-17 mean fluorescence intensity (MFI) (**Fig 6B**). Furthermore, addition of rIL-1α also amplified mRNA (**Fig 6C**) and protein levels of IL-17 (**Fig 6D**), showing that IL-1α boosts IL-17 production by CD4^+^ T cells that are already committed to the Th17 subset. Because IL-1 signaling promotes T cell proliferation [39] we analyzed the expansion capacity of IL-1α-treated Th17 cell by evaluating the cellular proliferation ratio of CFSE-labeled cells after 5 days of anti-CD3 plus anti-CD28 stimulation. Th17 committed CD4^+^ T cells incubated with IL-1α displayed greater cellular division compared to conventional Th17 cells (**Fig 6E**). This suggests that the reduced frequency and number of CD4^+^ IL-17^+^ T cells in the lung of infected *Il1a*^-/-^ animals are affected by an impaired proliferative capacity.

Beyond increasing cellular proliferation and IL-17 production, IL-1α could induce a molecular reprogramming of Th17 cells to enhance their effector functions. To address this question, we profiled the expression of Th17-related genes in naïve CD4^+^ T cells (Th0), conventional Th17 cells and conventional Th17 cells + IL-1α. Principal component analysis (PCA), including the whole array of genes, demonstrates that each of the conditions acquired distinct transcriptional profiles (**Fig 6F**). Furthermore, the direct comparison between the three conditions revealed the differential expression of several genes. By grouping 20 of these genes based on their biological functions, we observed that genes associated with Th17 differentiation such as *Il1r1*, *Il23r*, *Il6ra*, *Il23r*, and *Rorc* were equally expressed by conventional Th17 cells and IL-1α-treated Th17 cells (**Fig 6G**). However, IL-1α upregulated *Ccr6*, *Ifng* and *Tgfb1* expression (**Fig 6H**), while downregulating the expression of genes coding for IL-17 inhibitors, such as *Socs1* and *Il27* (**Fig 6I**). Notably, IL-1α downregulated the expression *Stat4*, *Il7r*, *Il10*, and *Icos*, while upregulating *Il9* (**Fig 6H**). Taken together, these results demonstrate that IL-1α promotes transcriptional and functional adaptations of Th17 cells. Of note, we observed that IL-17 enhances IL-1α production by BMDMs infected with *P*. *brasiliensis* (**Fig 6J**). To validate that the decreased Th17 response is responsible for the increased susceptibility of IL-1α deficient mice, we treated *P*. *brasiliensis*-infected *Il1a*^-/-^ mice intranasally with 500 ng of rIL-17A. We found that the protective immunity was restored by IL-17A treatment, which significantly reduced the pulmonary fungal load when compared to untreated *Il1a*^-/-^ mice at 30dpi (**Fig 6K**). This indicates that IL-α reprograms Th17 lymphocytes to produce high levels of IL-17, which promote an inflammatory loop by sustaining IL-α production and enhancing effector antifungal mechanisms by innate cells. Taken together, these results demonstrate that IL-1α is released via IFN-β/caspase-11 axis to shape Th17 immunity and resistance against *P*. *brasiliensis* infection.

## Discussion

Murine caspase-11 was discovered in 1993 by Yuan et al. [40] owing on its similarity to caspase-1. The 5'UTR promoter region, which regulates the expression of caspase-11 gene, has multiple NF-κB-binding sites but only a single binding site for STAT [41]. It has been reported that caspase-11 is activated, independently of TLR4 signaling [42, 43], mainly in response to the lipid A contained in the LPS from gram-negative bacteria [32, 42–45]. Compelling findings with *Candida albicans* [46] and *Aspergillus fumigatus* [47] demonstrate that the activation of the non-canonical caspase-11 inflammasome pathway is not limited to gram-negative bacteria. In a model of candidiasis, the host cell recognizes, engulfs and phagocytizes the fungus. While the phagosome matures, the fungus senses and responds to entry into the host cell initiating filamentation and cell wall biosynthesis [48, 49]. The remodeling of its surface exposes moieties, mainly ergosterol, capable of inducing macrophage lysis via inflammasome activation [50, 51]. Our study highlights the importance of caspase-11 in a non-bacterial infection, integrating the IFN-β/caspase-11 axis with IL-1α release and resistance to *P*. *brasiliensis* challenge. We have demonstrated that *P*. *brasilensis*-induced IFN-β activates caspase-11 to release IL-1α through a gasdermin D-dependent cell death. Nonetheless, we cannot discard that ergosterol, one of the most prevalent lipids found in *P*. *brasiliensis* vesicles [52–54], is also involved in the macrophage toxicity.

Caspase-11 induces cell death by promoting the proteolysis of the 53 kDa protein gasdermin D. Cleavage of human and mouse gasdermin D at Asp275 residue generates a 31 kDa N-terminal fragment that oligomerizes and forms ~20nm pores in the plasma cell membrane [19, 20, 27, 28], which become permeable to low molecular weight dyes such as EtBr and PI [55, 56]. Caspase-11 was required for EtBr and PI incorporation and LDH release by *P*. *brasiliensis*-infected macrophages, indicating pyroptotic cell death. The death of *P*. *brasiliensis*-infected cells allows the leakage of IL-1α in a caspase-11 and gasdermin D-dependent manner. The lysis of macrophages is a process that has also been widely observed for the opportunistic pathogenic fungus *C*. *albicans* [57, 58]. Caspase-11-mediated pyroptosis seems to be actively induced by *P*. *brasiliensis*, because dead fungal cells were unable to stimulate this process in macrophages. This could also mean that dead yeasts lack molecules that are recognized by innate immune receptors and necessary to trigger this pathway. Likewise, it has been reported that the lysis of macrophages also requires live *C*. *albicans* that forms hyphae, which stress phagosomal membranes [59], disrupt the macrophages with their associated toxin [57] and compete with the host for glucose [60]. In this context, a pertinent question that comes out of these findings is whether the induced pyroptosis benefits the host, the fungus or both. For instance, activation of pyroptosis in response to *Candida* might serve to augment proinflammatory responses, but *C*. *albicans* hijacks host pathways of programmed cell death for macrophage killing and evasion of the immune response [61]. However, considering that impaired pyroptosis in *Casp11*^-/-^ macrophages correlates with elevated fungal burden *in vitro* and *in vivo*, our study provides evidence for caspase-11-mediated pyroptosis as a critical process for resistance against the infection.

Endogenous danger signals released due to caspase-11-mediated pyroptotic death could activate the NLRP3 inflammasome and IL-1β/IL-18 production, a platform that plays a protective role during experimental PCM [30]. Because pore formation usually happens earlier than IL-1β production and processing, pyroptosis has been proposed as a mechanism by which IL-1β is secreted [18]. Nevertheless, our data do not support this hypothesis. Even though infected *Casp11*^-/-^ macrophages displayed reduced pore formation in cell membranes, IL-1β levels were still detected *in vitro* and *in vivo*. This suggests that pore formation is not an essential prerequisite for IL-1β secretion. Corroborating this concept, intact IL-1β processing and release in caspase-11-deficient mice was also reported in aspergillosis [47]. However, it is still possible that pores formed in response to caspase-1 activation in *Casp11*^-/-^ cells are enough for IL-1β secretion. Greater susceptibility of *Casp11*^-/-^ cells to *P*. *brasiliensis* infection was associated with reduced IL-1α production, because the treatment of *Casp11*^-/-^ mice with recombinant IL-1α protected the mice from fungal replication when compared to non-treated knockout mice. Nevertheless, diminished levels of pulmonary IL-1α in *Casp11*^-/-^ mice did not correlate with reduced survival, once these animals exhibited mortality rates comparable to that of WT mice after *P*. *brasiliensis* challenge. Because the production of IL-1α was not completely abrogated in *Casp11*^-/-^ mice, we speculate that alternative mechanisms [62] could mediate the release of this cytokine during *P*. *brasiliensis* infection. Modest IL-1α levels in *Casp11*^-/-^ mice might attenuate mortality, but they are not sufficient to control fungal growth in the lung. Moreover, studies with *A*. *fumigatus* have suggested that caspase-11 induces actin-mediated phagosomal killing to control fungal dissemination *in vivo* [63, 64]. Therefore, caspase-11 might also regulate IL-1α-independent pathways and interfere with fungal colonization and growth in other organs. Further studies are needed to confirm this hypothesis.

In contrast to IL-1β, unprocessed IL-1α is also active, but pro-IL-1α can be cleaved by calpains [65, 66], independently of caspase-1 activity, in a C-terminal fragment with a calculated mass of 18kDa [12]. Our study demonstrates that caspase-11 coordinates IL-1α release in response to *P*. *brasiliensis*, whereas IL-1β maturation and secretion depends on different molecular pathways [30, 31]. However, we have not addressed whether IL-1α was cleaved in the supernatant or the cell lysates after *P*. *brasiliensis* infection. Because many activators of IL-1α secretion, described by Tschopp group [12], are inducers of Ca^2+^ influx and patients with disseminated PCM present hypercalcemia [67], we suppose that calpain-like proteases can also influence IL-1α cleavage after *P*. *brasiliensis* stimulation. Given that IL-1α and IL-1β bind and activate the same receptor [13], they should in principle have identical biological functions. However, both IL-1 cytokines exert non-redundant roles on the host immune response against different pulmonary pathogens such as *A*. *fumigatus*, *Histoplasma capsulatum* and *Cryptococcus neoformans* [23, 68, 69]. In systemic candidiasis, for example, IL-1α increases leukocyte antifungal activity, while IL-1β recruits neutrophils [24]. Herein, we showed that IL-1α-deficiency led to exacerbated *P*. *brasiliensis* replication and a non-resolving granulomatous inflammation. Accordingly, our previous work revealed that, *P*. *brasiliensis*-infected *Il1r1*^-/-^ animals harbor more yeasts inside organized and compact granulomas [30]. These similarities with IL-1α-knockout mice indicate a prominent role for IL-1α in activating IL-1RI signaling. Nonetheless, we do not discard that these events could also be mediated by IL-1β. Future research using *Il1b*^-/-^ and *Il1a*/*Il1b*^-/-^ mice will provide deeper understanding about what the role of IL-1β in PCM.

During respiratory tract infections, the early expression of IL-1α by CCR2^+^ monocytes precedes the recruitment of neutrophils to the lung [21, 23]. In addition, cytosolic extract of necrotic WT cells, but not from *Il1a*^-/-^ cells, induced recruitment of neutrophils via CXCL1/CXCR2 [70]. We found reduced neutrophil chemotaxis to the lung of infected *Il1a*^-/-^ mice. Equivalent to intratracheal LPS injection model [71], one possibility is that IL-1α absence prevents tissue extravasation of the circulating neutrophils as a result of the greater cellular adhesion to the vascular endothelium [72]. However, our data point to a major role for IL-1α in shaping antifungal Th17 immunity, which boosts IL-17 production to regulate neutrophil recruitment into sites of infection. We have found that the treatment of *Casp11*^*-/-*^ mice with rIL-1α not only reduced CFU counts, but also increased IL-17 levels in the lung. Furthermore, macrophages increase the expression of IL-17A receptor in response to an inflammatory stimulus [73], while we showed that IL-17 amplifies the production of IL-1α by *P*. *brasiliensis*-infected macrophages and reverses the susceptibility of *Il1a*^-/-^ mice to *P*. *brasiliensis* infection.

Dendritic cells (DCs) infected with *Leishmania amazonensis* preferentially drive CD4^+^ T cells to an IFN-γ^low^IL-17^high^ profile, because they secrete more IL-1α than IL-1β, besides being weak IL-12p40 producers [74]. Following this logic, M-CSF-differentiated macrophages expressing membrane-bound IL-1α converts IL-17^-^ CD4^+^ T memory cells into conventional Th17 cells [75]. Nonetheless, IL-1α-deficiency did not affect the intrinsic capacity of naïve CD4^+^ T cells to differentiate into Th17 cells. Instead, we observed that IL-1α favors Th17 cell expansion, correlating with reduced amounts of Th17 cells in the lungs of infected *Il1a*^*-*/-^ mice. Previous study showed that long-term Th17 cells are maintained by IL-1 [76], while these cells are completely absent in IL-1R knockout mice [77]. Thus, IL-1α promotes Th17 cell survival during infection, which is essential for the host immune defense against fungi [78].

Importantly, IL-1α also induces IL-6 expression and its suppression reduces IL-6 synthesis [79, 80]. In our murine model of *P*. *brasiliensis* infection, impaired IL-6 production seen in infected IL-1α-knockout cells could have a major effect on Th17 differentiation. However, it is important to note that the cytokine requirement for Th17 cells polarization differs substantially between mouse and human. The conditions that regulate the development of mouse Th17 cells *in vitro* and *in vivo* are well defined [81]. It is widely accepted that Th17 generation in mice is commonly induced by synergistic treatment with TGF-β and IL-6 [82–84]. While TGF-β is required for initiation, IL-6 promotes STAT3-mediated activation of RORγt as well as alleviates Foxp3-mediated inhibition of the Th17 program [82, 85]. However, IL-6 does not seem to be absolutely necessary for human Th17 cultures [86]. Alternatively, IL-21, activating STAT3, can act with TGF-β to generate human Th17 cells [87]. The cytokines IL-23 and IL-1β are most consistently found to play crucial roles in both mouse and human Th17 development [88–91]. The IL-1β dependency for Th17 differentiation has been already reported in *Candida* infection [91, 92]. Here, we demonstrate that IL-1α induces a transcriptional reprograming of cells already committed to the Th17 phenotype. In synergy with IL-23, IL-1β would control RORγt and IL-23R expression to sustain IL-17 production by effector Th17 cells [77, 88]. However, IL-1α seems to exert its effects independently of changes in RORγt and IL-23R abundance. Indeed, IL-1α suppresses the expression of inhibitory molecules, such as Socs1 and IL-27, and enhances the levels of TGF-β1 transcripts, a cytokine that (in mice) collaborates with IL-6 to promote T helper cell differentiation into Th17 profile [93]. Remarkably, Th17 cells stimulated with IL-1α simultaneously co-express IL-17A and IFN-γ, but not IL-10. Because *A*. *fumigatus*-exposed human PBMCs stop producing IL-10 to upregulate IL-17 [94], IL-1α possibly disrupts IL-10 /IL-17 balance, enabling IL-17 synthesis. Also, the flexibility of Th17 cells to acquire effector functions that are normally associated with Th1 responses such as IFN-γ production has been reported in *C*. *albicans* and *A*.*fumigatus* -primed cultures [91, 95]. Their shift to a Th1 cell-like phenotype has been linked to the pathogenicity of Th17 cells in colitis [96], arthritis [97], diabetes [98], and experimental autoimmune encephalomyelitis (EAE) [99]. According to Wang et al., 2014 [100], the development of IL-17/IFN-γ double producing cells is dependent on T-bet, Runx1, and Runx3 transcription factors. Nevertheless, the shift in cytokine production under IL-1α stimulation was not accompanied by an increase in Tbx21 mRNA levels. We speculate that the expression of IFN-γ by Th17 cells collaborates for effective antifungal immunity because IFN-γ is important for host resistance against fungal cells [101]. IFN-γ induces *P*. *brasiliensis*-infected macrophages to secrete TNF-α, required for the development and persistence of well-formed granulomas [102], and NO, which plays a well-documented role in fungal clearance [103].

Overall, this study discloses a pathway in which IL-1α is released due to caspase-11-dependent pyroptosis to enhance effector functions of innate cells and T lymphocytes. Further investigations shell reveal whether this molecular mechanism translates to human cell responses to *P*. *brasiliensis* and how it can be explored to design novel immunotherapeutic interventions.

## Materials and methods

### Mice

For experimental infection and isolation of bone marrow macrophages (BMDMs), six to seven-week-old male *Casp11*^-/-^ (kindly provided by Vishva Dixit, Genentech) and *Il1α*^-/-^ (kindly provided by Bernhard Ryffel, University of Orleans, Orleans, France) and strain-matched wild-type (C57BL/6) mice were obtained from the Isogenic Breeding Unit, Ribeirão Preto Medical School, University of São Paulo, Ribeirão Preto, Brazil. Mice were bred and maintained under specific pathogen-free conditions and provided with clean food and water ad libitum in the animal housing facility of the Department of Biochemistry and Immunology.

### Ethics statement

Mice experiments were conducted in compliance with the institutional guidelines on ethics for animal care approved by the Ethical Commission in Animal Research (approved protocol no. 218/2017), which follows the Brazilian National Guidelines recommended by the Brazilian Society of Science in Animals Laboratory.

### Fungus, in vivo infection and mortality

Yeast cells from a *P*. *brasiliensis-*virulent strain (Pb18) were cultured for 7 days at 37°C in brain heart infusion (BHI)-agar medium (Sigma) supplemented with gentamicin (96 μg/mL), and 5% fetal bovine serum (FBS) (Gibco). Pb18 cells were harvested and incubated overnight under agitation in F12 Coon's Modification medium (Sigma) at 37°C. Cell viability was determined by the fluorescein diacetate-ethidium bromide method, as previously described [104]. Only fungal suspensions containing more than 90% viable cells were used. For in vivo infection, six to seven-week-old male mice were anesthetized with ketamina (90mg/kg) and xylazine (5mg/kg) by intraperitoneal (i.p.) administration, followed by intravenous (i.v.) inoculation of 1x10^6^ yeast cells. The numbers of colony-forming units (CFU) in the organs were calculated at 15 and 30 days post-infection (dpi) by normalizing the count per gram of tissue. The survival of the Pb18-infected wild-type and knockout mice (10–12 mice of each group) was verified daily for 120 days.

### Pulmonary cytokine quantification by ELISA

Lungs from Pb18-infected mice, were removed, weighed, triturated in sterile PBS-containing protease inhibitor (Complete, Roche), and centrifuged. Supernatants were collected and stored at −20°C. Levels of IL-1α, IL-17A, IFN-γ and, IL-6 were measured by enzyme-linked immunosorbent assay (ELISA) according to manufacturers’ recommendations (BD Pharmingen). The reading was held in eMax ELISA reader (Molecular Devices) at 450 nm.

### Histopathological analysis

Animals selected at random from each group were euthanized at 15 and 30 days after infection. The lungs were excised, fixed with 10% formalin for 24 hours and embedded in paraffin. Tissue sections (5 μm) were stained with hematoxylin and eosin (H&E) for analysis of the lesions or impregnated with silver for fungal cell labeling (Gomori method) and reticulum fiber staining. The nuclei percentage and the integrated density of *P*. *brasiliensis* were estimated using Image J software.

### Lung cell isolation

Lungs from each mouse were excised, minced with scissors, and enzyme-digested at 37°C for 30–35 minutes in 1 mL of digestion buffer (2 mg/ml collagenase IV (Sigma), and 1 mg/ml DNase (Sigma)). Tissue fragments were further dispersed by repeated aspiration, crushed through a 50-μm pore size nylon filter (BD Biosciences) and then centrifuged (400 g, 10 min, 4°C). Erythrocytes in the cell pellets were lysed, and the remaining cells were resuspended in 5% RPMI. For pulmonary cell activation, 1x10^6^ cells/well were cultured for 4 h with PMA (50 ng/mL), ionomycin (500 ng/mL), and brefeldin A (5 mg/mL), followed by staining for extracellular and intracellular markers and analysis by flow cytometry.

### CD4^+^ T cell isolation for Th17 cell differentiation and proliferation

CD4^+^ T cells from spleens and lymph nodes of 5-7-week old C57BL/6 mice were purified by magnetic separation using anti-APC microbeads (Miltenyi Biotec). For Th17 differentiation, the cells were activated for 5 days by plate- bound anti-CD3 (2 μg/ml) and anti-CD28 (1μg/m) in the presence of 5 ng/mL TGF-β, 20 ng/mL IL-6, 50 ng/mL IL-23, 10 ug/mL anti-IL-2, 10 ug/mL anti-IFN-γ and 10 ug/mL anti-IL-4. All recombinant cytokines were obtained from R&D and neutralizing antibodies from BioXCell. When indicated, 10 ng/mL of IL-1α was added to the cell culture after 3 days of differentiation. Differentiated Th17 cells were stained with CFSE and lymphocyte proliferation was monitored by flow cytometry.

### Flow cytometry

Cells were stained with fluorochrome-conjugated antibodies specific for the surface molecules CD3, CD4, MHCII, CD11c, CD11b, Ly6G, CCR6 and for the intracellular cytokines IL-17 and IFN-γ. For the intracellular cytokine staining, cells were previously permeabilized using PBS containing 1% FBS, 0.1% sodium azide and 0.2% saponin. Data acquisition performed using a FACSCanto II flow cytometer (BD Biosciences) were plotted and analyzed using FlowJo software.

### Immunohistochemistr

Lungs from C57BL/6 and *Il1a*^-/-^ mice were immersed in OCT medium (Sakura Finetek), snap-frozen in liquid nitrogen, and stored at −80°C until analysis. Frozen tissue sections (5 μm) were fixed with acetone and endogenous peroxidase activity and non-specific sites were blocked with 3% hydrogen peroxide and with PBS plus 3% (W/V) non-fat milk, respectively. Following, the slides were incubated with rat IgG anti-mouse Ly6G (Biolegend) or IgG anti-mouse (as control) antibodies before the incubation with biotin-labeled antibody (Vector Laboratories). Staining was developed with 3,3'-diaminobenzidine (Vector Laboratories) and the reaction was counterstained with Mayer’s hematoxylin. We measured immunostained areas using Image J software. Briefly, the range of positivity was defined using the IHC Tool box. Next, the images were converted to 8-bit, and the grayscale was converted to binary (black and white). The threshold was adjusted, and the labeled areas became the black portions. Finally, the percentage of stained area was analyzed.

### Quantitative Real-Time Polymerase Chain Reaction (qPCR)

Total RNA was extracted from mouse tissue using the TRIzol reagent (Invitrogen) and the SV Total RNA Isolation System Kit (Promega) according to the manufacturer’s instructions. Complementary DNA was synthesized through a High Capacity cDNA Reverse Transcription Kit (Applied Biosystems). SYBR Green Mix–based real-time quantitative PCR assays were performed using the StepOnePlus Real-Time PCR System (Applied Biosystems). The mean cycle threshold (Ct) values of triplicate measurements were used to calculate the expression of the target genes, which were normalized to the housekeeping gene β2m and analyzed with the 2^–ΔΔCt^ method. Primers (presented in S1 Table) were designed using the Primer Express software package v2.0 (Applied Biosystems), based on the nucleotide reference sequences available at GenBank database.

### PCR array

The 96-well precoated plate (PAMM-073Z; Qiagen) together with the SYBR Green Quantitative PCR Master Mix (SAbiosciences) was used to examine by qPCR a set of 89 Th17 response related genes in samples of CD4^+^ T cells cultured under the following three conditions: Th0, Th17 and Th17 plus IL-1α. The samples from four different wells were polled together to obtain enough RNA for the whole array. RNA integrity was analyzed by Agilent Bioanalyzer following the manufacturer’s instructions. The raw data were normalized to the cycling threshold (Ct) from the housekeeping gene Gusb (β-glucuronidase) and analyzed with the 2^–ΔCt^ method. Principal component analysis (PCA) and heat maps were generated using the R Language and Environment for Statistical Computing v.3.5.0 and the packages *ggplot2* and *gplots*.

### In vivo treatment protocols

Where indicated, *Casp11*^-/-^ mice were treated intranasally (i.n.) on days 0, 1, 4, 7, 14, 21 and 28 with 100 ng/mice of recombinant mouse IL-1α (rIL-1α; R&D Systems) and were killed on day 30 post *P*. *brasiliensis* infection for analysis of CFU and cytokines in the lung. Where indicated, every three days from days 15 to 27 after *P*. *brasiliensis* challenge, recombinant mouse IL-17A (rIL-17A; Gibco) was administrated i.n. at a dose of 0.5 ug per mouse in a total of 25 uL. The pulmonary fungal burden was determined at day 30 post infection.

### Bone marrow-derived macrophages

Bone marrow-derived macrophages (BMDM) were differentiated from femurs of 7-week-old WT, *Il1a*^-/^-, and *Casp11*^-/-^ naïve mice, by culturing precursor cells in RPMI 1640 medium supplemented with 20% FBS and 30% L929 cell–conditioned media for 7 days at 37°C and 5% CO_2_, as previously described [105]. After differentiation, cells were harvested, seeded and infected with *P*. *brasiliensis* at MOI (multiplicity of infection) of 5. IL-1β and IL-1α levels in culture supernatants were measured 48 hours post-infection using standard sandwich ELISAs according to the manufacturer’s recommendations (BD Biosciences). When indicated, BMDMs were treated with 5 or 10 ug/mL of neutralizing IFN-β antibody (PBL Assay Science) at the same time of the infection; or pre-treated with or without recombinant IFN-β (100 or 1000 U/mL) 2 hours before *P*. *brasiliensis* infection; or incubated for 6 h with 50 or 100 ng/mL of recombinant IL-17A (BioSource-Life Technologies) before infection.

### Western blotting

BMDMs (1x10^6^ cells/well) were infected with viable fungi at a MOI of 5 in the indicated time points. Cell-free supernatants and cell lysates were collected and subjected to western blotting. Primary antibodies included a monoclonal rat anti-caspase-11 (Sigma) and a goat anti-mouse IL-1β (Sigma). In some cases, proteins from the cell culture supernatants were precipitated with methanol-chloroform.

### Pore formation assay

In this assay, fungus was added at a MOI of 5 to 2x10^5^ BMDMs plated on 13-mm glass coverslips in 24-well tissue culture dishes. After 24 hours of incubation at 37°C in 5% CO_2_, the coverslips were washed and then inverted onto a 5uL of PBS containing 25 μg/mL EtBr and 5 μg/mL acridine orange. Images were acquired using a fluorescence microscope (Olympus America) and analyzed with the ImageJ software. Pore formation was calculated according to the percentage of permeable cells to EtBr that were photographed (10x objective) from three different fields containing approximately 400 cells/field.

### Lactate dehydrogenase release assay

BMDMs were seeded into 24-well plates (2x10^5^ cells/well) and cultured in RMPI 1640 media lacking phenol red with 15mM HEPES and 2g/L NaHCO3 supplemented with 10% FBS. Lactate dehydrogenase (LDH) released by 24-hour *P*. *brasiliensis*-infected macrophages was quantified in culture supernatants using the CytoTox 96 LDH-release kit (Promega). Percentage of LDH release was calculated as follows: (LDH infected—LDH uninfected/LDH total lysis—LDH uninfected x 100). LDH total lysis was determined by lysing the cultures with Triton X-100.

### Detection of pyroptosis by propidium iodide incorporation

BMDMs (10^6^ cells/well) were infected with *P*. *brasiliensis* at a MOI of 5. After 24 h of incubation, the frequency of macrophages undergoing pyroptosis cell death was defined by the loss of membrane integrity evaluated by propidium iodide (PI) staining. Data were acquired on a FACSCanto II flow cytometer (BD Biosciences) and analyzed using FlowJo software (Tree Star, Ashland, OR).

### Killing assay and NO detection

BMDMs were plated on 96 wells culture plates at 2x10^5^ cells/well, pre-treated for 6 hours with 10 or 50 ng/mL of recombinant IL-1α (rIL-1α; R&D Systems) or overnight with 50 ng/mL of recombinant IFN-γ (rIFN-γ; BD Biosciences), infected with *P*. *brasiliensis* (MOI 1:25 fungi per macrophages) and incubated at 37°C, 5% of CO_2_ for 2 hours. After this period, extracellular yeast cells were removed by washing the wells, and cells were cultured for 48 hours. Finally, cells were lysed with saponin (0.05%) for colony forming unit (CFU) counting. Nitrite production in the culture supernatants was estimated using the Griess reaction in BMDM cultures stimulated at a MOI of 5, as described previously [106].

### Statistical analysis

Data are expressed as means ± SD. For comparison between multiple groups, one-way ANOVA followed by the Tukey-Kramer post-test was applied. The log-rank test (Mantel–Cox) was used to compare survival curves and unpaired parametric student´s t-test was used to compare differences between two groups that assumed a normal distribution. Comparisons between two experimental groups not normally distributed were performed with non-parametric Mann-Whitney U statistical test. The normal distribution of data was analyzed by D’Agostino test. All analyzes were performed using the Prism 5.0 software (GraphPad Software). P values < 0.05 were considered statistically significant.

## Supporting information

S1 Table Murine primer sequences used in the study. (DOCX)

S1 Fig *P. brasiliensis*-driven IL-1β production is sustained even in the absence of caspase-11. **(A)** IL-1β production by WT and *Casp11*^-/-^ macrophages at different lengths of time post *P*. *brasiliensis* infection. **(B)** IL-1β was quantified in lung homogenates from WT and *Casp11*^-/-^ mice 15 and 30 days after intravenous infection with *P*. *brasiliensis*. Data are representative of three independent experiments expressed as the mean of triplicate wells. Statistical analysis was performed using or parametric Student’s t test (B– 15dpi) or non-parametric Mann-Whitney U test (B– 30 dpi). Error bars depict ± SD. ns: not significant.
(TIF)

S2 Fig *P. brasiliensis* yeasts are located within compact granulomas contoured by a dense reticulin ring. **(A)** Histological slides, stained with hematoxylin and eosin, were prepared from WT and *Il1a*^-/-^ animals at 15 and 30 days after infection by *P*. *brasiliensis*. The images were taken using a light microscope (magnification of 200x) and the inflammatory infiltrate was quantified. **(B)** At 30 days of infection, the presence, formation and organization of reticulin fibers in the lung of WT and *Il1a*^-/-^ mice were analyzed by the Gomori method (magnification of 200x). Results are representative of two experiments. Statistical analysis was performed using non-parametric Mann-Whitney U test (A-15dpi) or parametric Student’s t test (A-30dpi). dpi: days post infection.
(TIF)

S3 Fig IL-1α contributes to fungi elimination by enhancing NO production. **(A)** BMDMs from wild-type or IL-1α knock-out mice were pre-activated for 16 hours with recombinant IFN-γ (rIFN-γ; 50 ng/mL) followed by infection with *P*. *brasiliensis* in a yeast:macrophage ratio of 5∶1. After 48 hours of infection, the levels of nitrite in the culture supernatant were measured using the Griess assay and ELISA, respectively. **(B)** Fungal load in BMDMs from WT or *Il1a*^-/-^ mice stimulated with rIFN-γ for 16 hours. **(C)** WT and *Il1a*^-/-^ mice were inoculated with 1x10^6^ yeasts of *P*. *brasiliensis*. The expression of NOS2 was evaluated in the pulmonary tissue by qPCR at 30dpi. The results are representative of three independent experiments performed in triplicate. Statistical analysis was performed using one-way ANOVA with Tukey’s multiple comparison test (A-B) and non-parametric Mann-Whitney U test (C). (*) p < 0.05, compared with IL-1α-deficient cells. (#) p< 0.05, compared with WT cells cultured in the presence of IFN- γ.
(TIF)

S4 Fig *Casp11*^-/-^ mice infected with *P. brasiliensis* exhibit decreased Th17 response pattern in the lung. WT and *Casp11*^-/-^ mice were infected with virulent yeast strain 18 of *P*. *brasiliensis* (Pb18) for 30 days (1x10^6^ cells, i.v.). (**A-B**) IL-17 transcripts were measured by quantitative PCR, while IL-17 production was evaluated with ELISA. **(C)** Cell suspension isolated at 30 dpi from the lungs of Pb18-infected WT and *Casp11*^-/-^ mice were stimulated with PMA and ionomycin for 4 hours before the frequency and absolute number of IL-17A-producing T CD3^+^CD4^+^ cells were assessed by flow cytometry. **(D)** Measurement of IL-17 production in the lung homogenate from *Casp11*^-/-^ mice treated or not with rIL-1α at the beginning of *P*. *brasiliensis* infection. Results are representative of three independent experiments. Statistical analysis was performed using non-parametric Mann-Whitney U test (A-C) and one-way ANOVA with Tukey’s multiple comparison test (D). Bars represent the mean ± SD of 5 mice. (*) p < 0.05 compared to WT control mice. (&) indicates p <0.05 compared with non-treated *Casp11*^-/-^ mice.
(TIF)

S5 Fig IL-17-producing T cells migrate toward the lung in IL-1α-deficient mice. **(A)** After infecting wild-type (WT) and IL-1α-deficient mice with 10^6^ yeasts cells of *P*. *brasiliensis* for 30 days, leukocytes derived from mediastinal lymph nodes and lungs were used to evaluate the frequency of CD3^+^CD4^+^IL-17A^+^ cells. For **(B)** mRNA expression and **(C)** protein quantification of CCL20, lungs from WT and *Il1a*^-/-^ mice infected by *P*. *brasiliensis* were harvested at 30 dpi **(D)** CCR6^+^-expressing Th17 cells in the lung of *P*. *brasiliensis*-infected WT and *Il1a*^-/-^ mice at 30 dpi. Data were plotted as frequency of positive cells and MFI. Data represent mean ± SD of five mice per group. Results are representative from two independent experiments. Statistical analysis was performed using non-parametric Mann-Whitney U test (B and D) or parametric Student’s t test (C). MFI: median of fluorescence intensity.
(TIF)

S6 Fig IL-1α is dispensable for Th17 differentiation. **(A)** Naïve T cells from *Il1a*^-/-^ mice were activated with anti-CD3 and anti-CD28 and differentiated into Th17 cells from 5 days before analysis of cytokines by flow cytometry. The Th17 differentiation of WT cells is shown in Fig 6A. **(B)** Production of IL-17 by naïve WT and *Il1a*^-/-^ CD4^+^ T cells culture for 5 days under neutral (Th0) or Th17-polarizing conditions. **(C)** *Ifng* expression assessed with RT-qPCR in IL-17-secreting CD4^+^ T cells treated or not with IL-1α on the day 3 of *in vitro* Th17 differentiation. **(D)** IFN-γ produced by Th17 cells cultured or not with IL-1α from the third day was quantified on the 5^th^ day of incubation with ELISA. The results are representative of three independent experiments performed in triplicate. Statistical analysis was performed one-way ANOVA with Tukey’s multiple comparison test (B). (*) p <0.05 comparing WT Th0 and Th17 cells. ns: not significant.
(TIF)
