# Supplementary material for: Caspase-11-dependent IL-1α release boosts Th17 immunity against Paracoccidioides brasiliensis
Source: PLoS Pathog. 2019 Aug 19;15(8):e1007990. doi: 10.1371/journal.ppat.1007990 (PMC6715237; doi:10.1371/journal.ppat.1007990)
Supplement: S1 Table — (DOCX) [file ppat.1007990.s001.docx]

| Gene | Forward (5'-3') | Reverse |
| --- | --- | --- |
| *Β2m* | CACCCCCACTGAGACTGATACATA | TCACATGTCTCGATCCCAGTAGA |
| *Ifng* | CATGGCTGTTTCTGGCTGTTAC | CCAGTTCCTCCAGATATCCAAGA |
| *Il17a* | GGACTCTCCACCGCAATGA | GCACTGAGCTTCCCAGATCAC |
| *Il6* | AAGAAAGACAAAGCCAGAGTCCT | TTTTACCTCTTGGTTGAAGATATGAA |
| *Ccl20* | AACCTCCTCAGCCTAAGAGTCAAG | CAGCTGTGATCATTTCCTCCTT |
| *iNOS* | CGAAACGCTTCACTTCCAA | TGAGCCTATATTGCTGTGGCT |
|  |  |  |
