# Supplementary figures and images for: Caspase-11-dependent IL-1α release boosts Th17 immunity against Paracoccidioides brasiliensis
Source: PLoS Pathog. 2019 Aug 19;15(8):e1007990. doi: 10.1371/journal.ppat.1007990 (PMC6715237; doi:10.1371/journal.ppat.1007990)

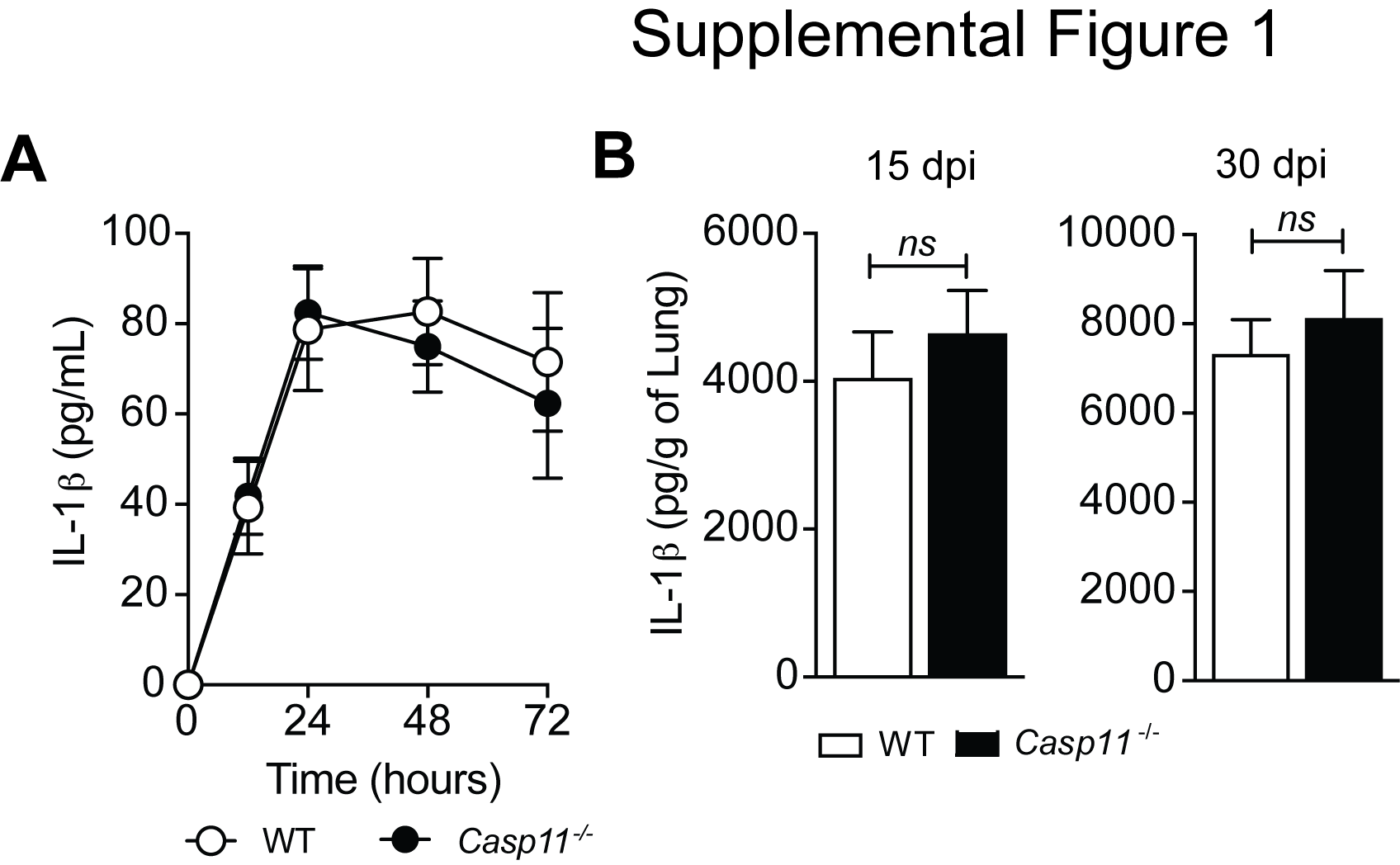

Supplement: S1 Fig — (A) IL-1β production by WT and Casp11-/- macrophages at different lengths of time post P. brasiliensis infection. (B) IL-1β was quantified in lung homogenates from WT and Casp11-/- mice 15 and 30 days after intravenous infection with P. brasiliensis. Data are representative of three independent experiments expressed as the mean of triplicate wells. Statistical analysis was performed using or parametric Student’s t test (B– 15dpi) or non-parametric Mann-Whitney U test (B– 30 dpi). Error bars depict ± SD. ns: not significant. (TIF) [file ppat.1007990.s002.tif]

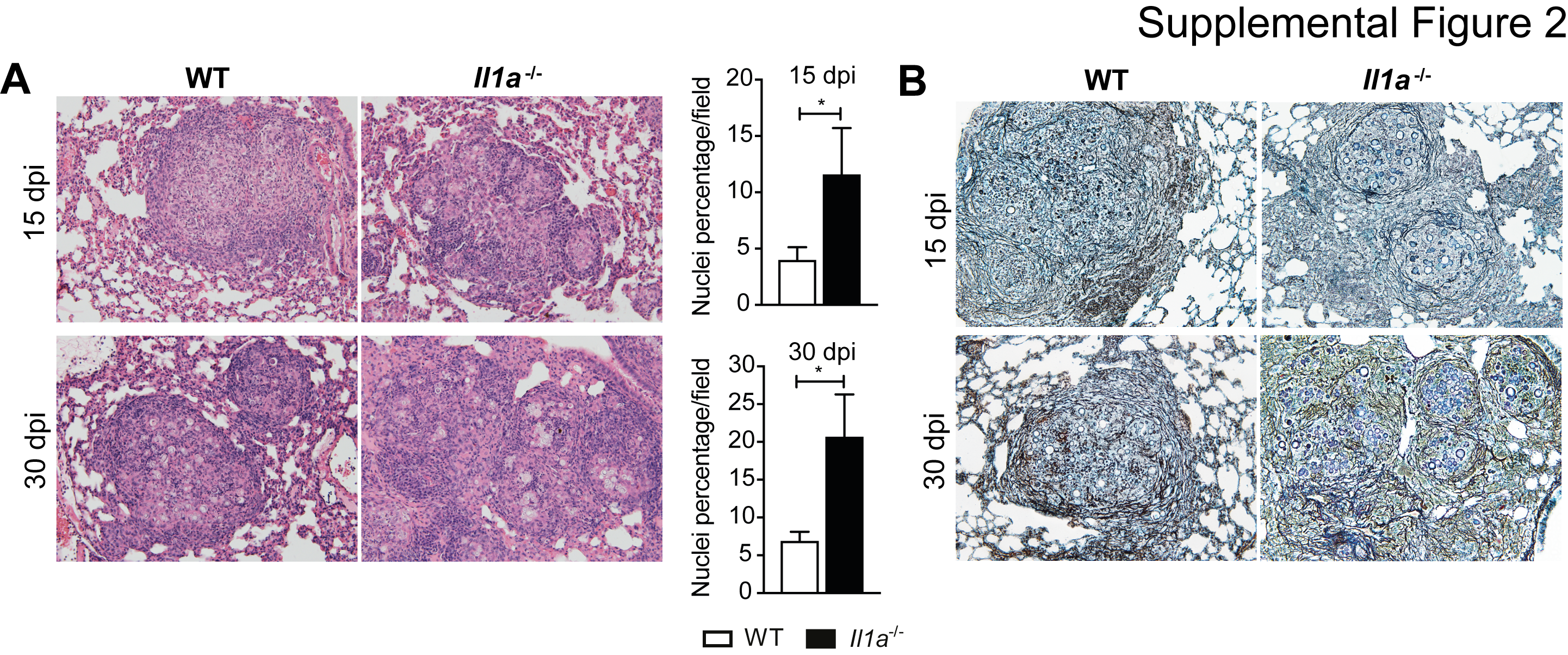

Supplement: S2 Fig — (A) Histological slides, stained with hematoxylin and eosin, were prepared from WT and Il1a-/- animals at 15 and 30 days after infection by P. brasiliensis. The images were taken using a light microscope (magnification of 200x) and the inflammatory infiltrate was quantified. (B) At 30 days of infection, the presence, formation and organization of reticulin fibers in the lung of WT and Il1a-/- mice were analyzed by the Gomori method (magnification of 200x). Results are representative of two experiments. Statistical analysis was performed using non-parametric Mann-Whitney U test (A-15dpi) or parametric Student’s t test (A-30dpi). dpi: days post infection. (TIF) [file ppat.1007990.s003.tif]

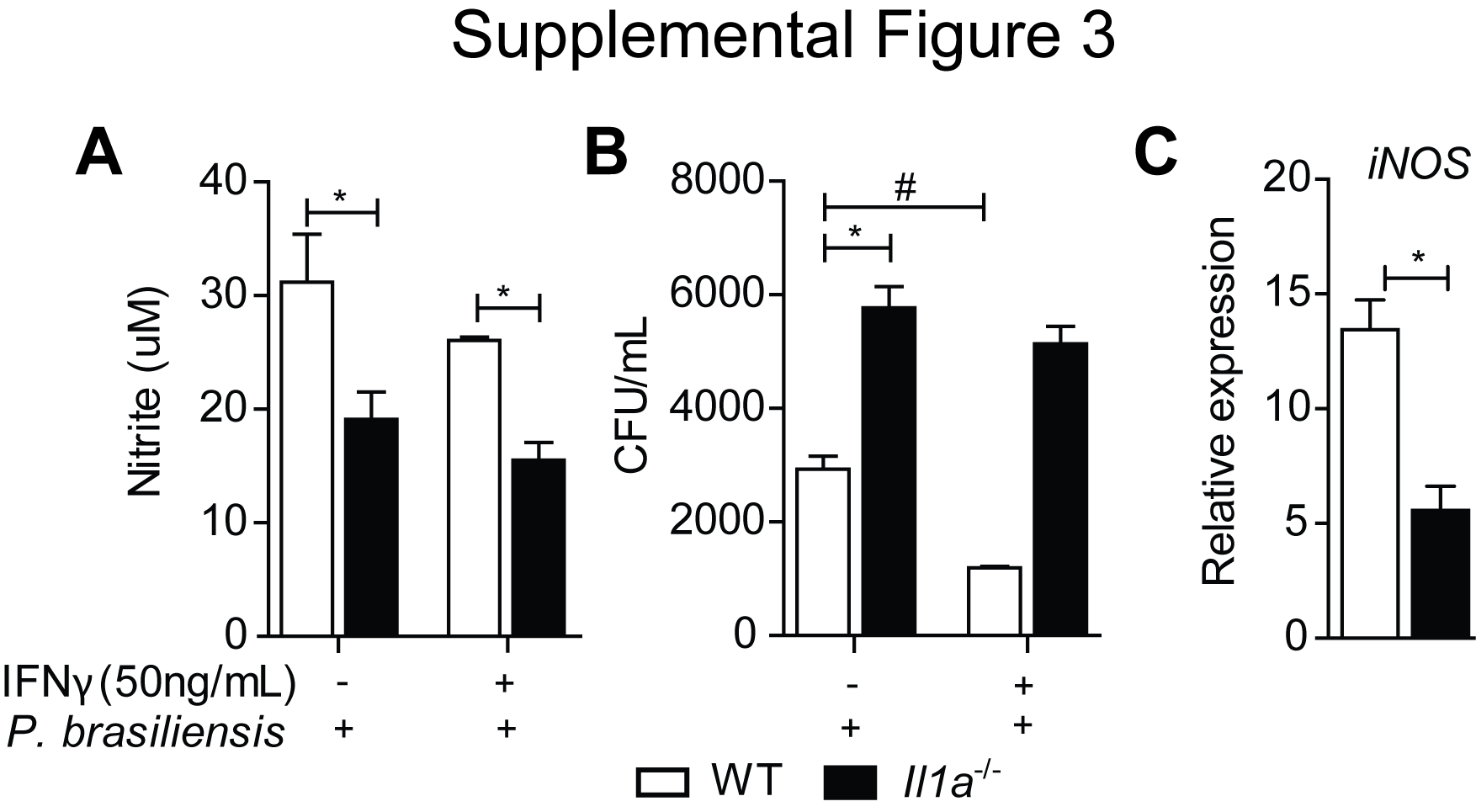

Supplement: S3 Fig — (A) BMDMs from wild-type or IL-1α knock-out mice were pre-activated for 16 hours with recombinant IFN-γ (rIFN-γ; 50 ng/mL) followed by infection with P. brasiliensis in a yeast:macrophage ratio of 5∶1. After 48 hours of infection, the levels of nitrite in the culture supernatant were measured using the Griess assay and ELISA, respectively. (B) Fungal load in BMDMs from WT or Il1a-/- mice stimulated with rIFN-γ for 16 hours. (C) WT and Il1a-/- mice were inoculated with 1x10^6^ yeasts of P. brasiliensis. The expression of NOS2 was evaluated in the pulmonary tissue by qPCR at 30dpi. The results are representative of three independent experiments performed in triplicate. Statistical analysis was performed using one-way ANOVA with Tukey’s multiple comparison test (A-B) and non-parametric Mann-Whitney U test (C). (*) p < 0.05, compared with IL-1α-deficient cells. (#) p< 0.05, compared with WT cells cultured in the presence of IFN- γ. (TIF) [file ppat.1007990.s004.tif]

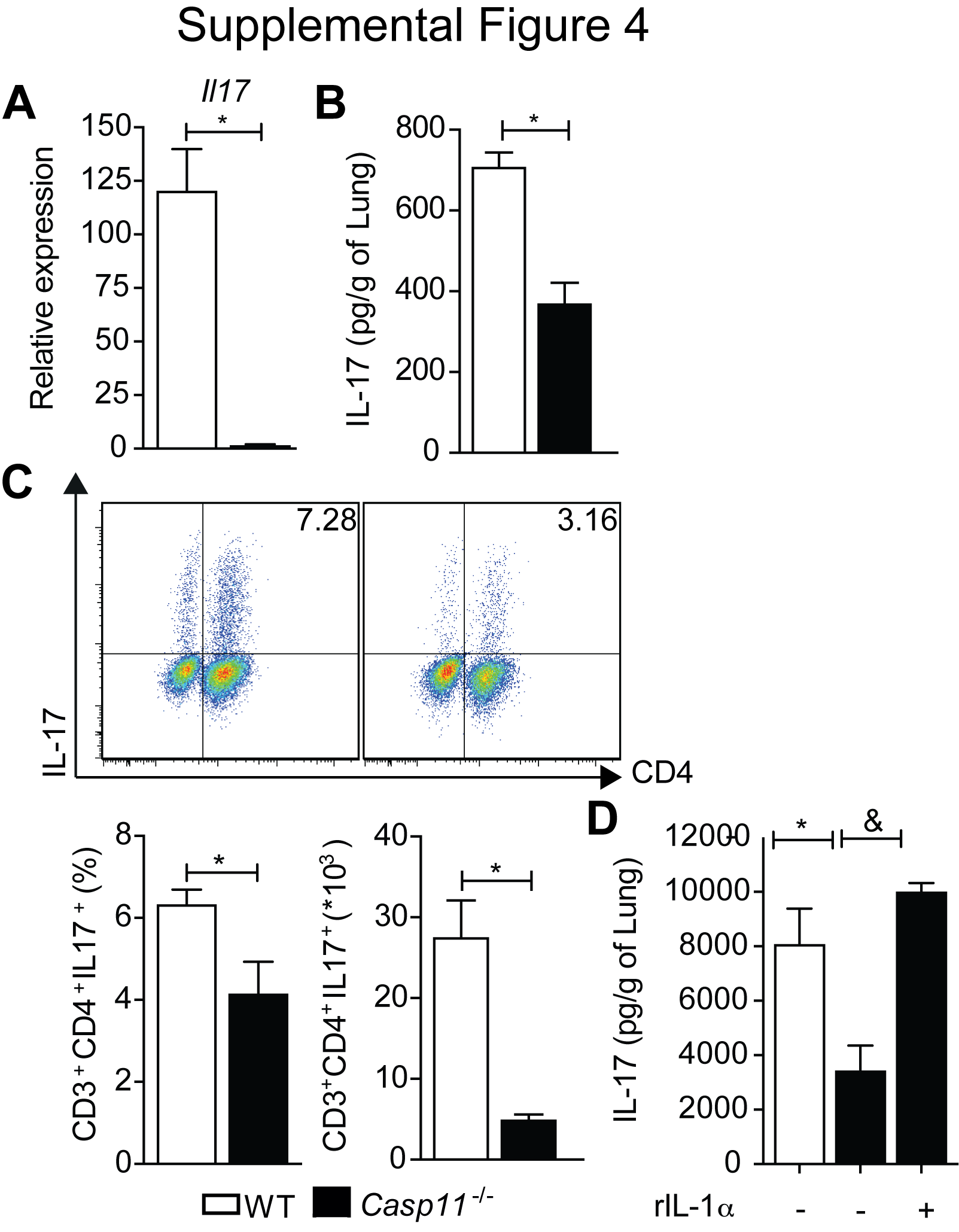

Supplement: S4 Fig — WT and Casp11-/- mice were infected with virulent yeast strain 18 of P. brasiliensis (Pb18) for 30 days (1x10^6^ cells, i.v.). (A-B) IL-17 transcripts were measured by quantitative PCR, while IL-17 production was evaluated with ELISA. (C) Cell suspension isolated at 30 dpi from the lungs of Pb18-infected WT and Casp11-/- mice were stimulated with PMA and ionomycin for 4 hours before the frequency and absolute number of IL-17A-producing T CD3+CD4+ cells were assessed by flow cytometry. (D) Measurement of IL-17 production in the lung homogenate from Casp11-/- mice treated or not with rIL-1α at the beginning of P. brasiliensis infection. Results are representative of three independent experiments. Statistical analysis was performed using non-parametric Mann-Whitney U test (A-C) and one-way ANOVA with Tukey’s multiple comparison test (D). Bars represent the mean ± SD of 5 mice. (*) p < 0.05 compared to WT control mice. (&) indicates p <0.05 compared with non-treated Casp11-/- mice. (TIF) [file ppat.1007990.s005.tif]

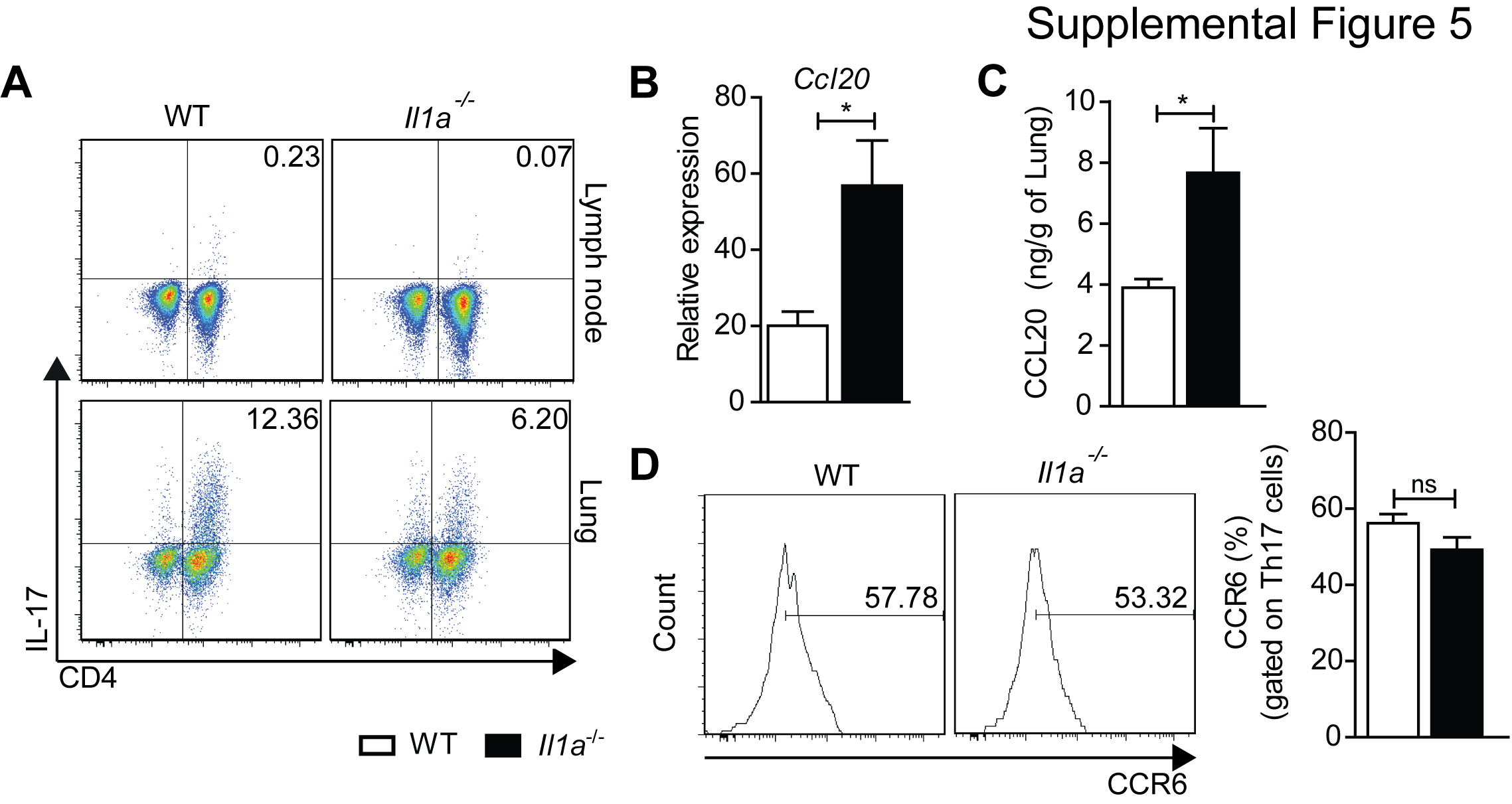

Supplement: S5 Fig — (A) After infecting wild-type (WT) and IL-1α-deficient mice with 106 yeasts cells of P. brasiliensis for 30 days, leukocytes derived from mediastinal lymph nodes and lungs were used to evaluate the frequency of CD3+CD4+IL-17A+ cells. For (B) mRNA expression and (C) protein quantification of CCL20, lungs from WT and Il1a-/- mice infected by P. brasiliensis were harvested at 30 dpi (D) CCR6+-expressing Th17 cells in the lung of P. brasiliensis-infected WT and Il1a-/- mice at 30 dpi. Data were plotted as frequency of positive cells and MFI. Data represent mean ± SD of five mice per group. Results are representative from two independent experiments. Statistical analysis was performed using non-parametric Mann-Whitney U test (B and D) or parametric Student’s t test (C). MFI: median of fluorescence intensity. (TIF) [file ppat.1007990.s006.tif]

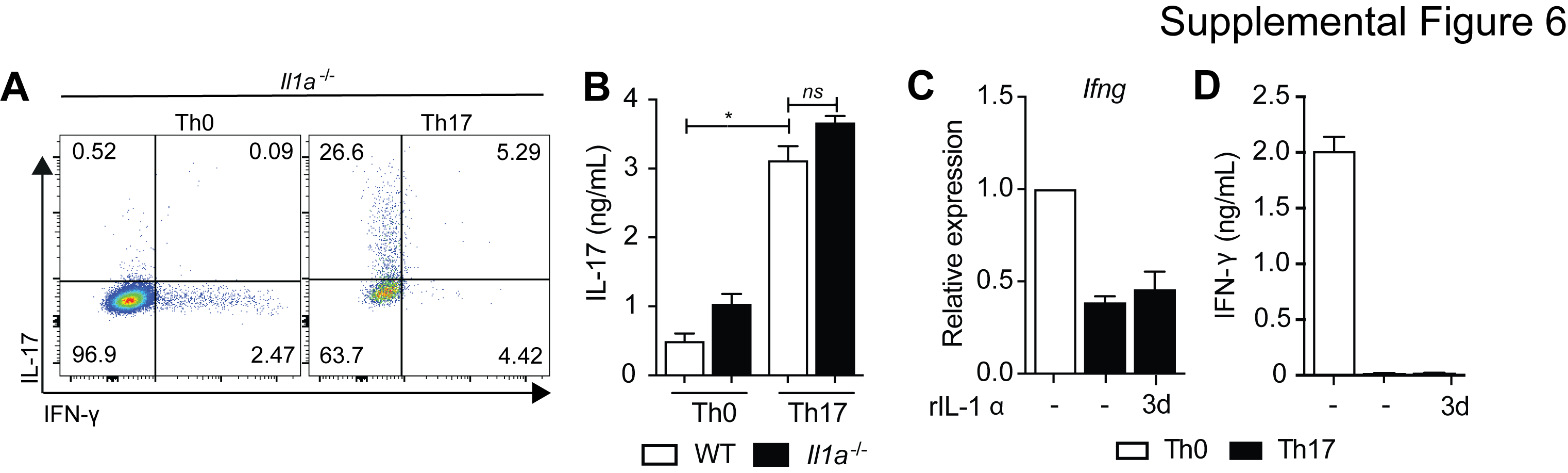

Supplement: S6 Fig — (A) Naïve T cells from Il1a-/- mice were activated with anti-CD3 and anti-CD28 and differentiated into Th17 cells from 5 days before analysis of cytokines by flow cytometry. The Th17 differentiation of WT cells is shown in Fig 6A. (B) Production of IL-17 by naïve WT and Il1a-/- CD4+ T cells culture for 5 days under neutral (Th0) or Th17-polarizing conditions. (C) Ifng expression assessed with RT-qPCR in IL-17-secreting CD4+ T cells treated or not with IL-1α on the day 3 of in vitro Th17 differentiation. (D) IFN-γ produced by Th17 cells cultured or not with IL-1α from the third day was quantified on the 5th day of incubation with ELISA. The results are representative of three independent experiments performed in triplicate. Statistical analysis was performed one-way ANOVA with Tukey’s multiple comparison test (B). (*) p <0.05 comparing WT Th0 and Th17 cells. ns: not significant. (TIF) [file ppat.1007990.s007.tif]
